# Analysis of the effects of bench-scale cell culture platforms and inoculum cell concentrations on PSC aggregate formation and culture

**DOI:** 10.3389/fbioe.2023.1267007

**Published:** 2023-12-01

**Authors:** Diepiriye G. Iworima, Robert K. Baker, James M. Piret, Timothy J. Kieffer

**Affiliations:** ^1^ Department of Cellular and Physiological Sciences, Life Sciences Institute, The University of British Columbia, Vancouver, BC, Canada; ^2^ School of Biomedical Engineering, The University of British Columbia, Vancouver, BC, Canada; ^3^ Michael Smith Laboratories, The University of British Columbia, Vancouver, BC, Canada; ^4^ Department of Chemical and Biological Engineering, The University of British Columbia, Vancouver, BC, Canada; ^5^ Department of Surgery, The University of British Columbia, Vancouver, BC, Canada

**Keywords:** human embryonic stem cells, bioreactor 3D cell culture, aggregation, expansion, seed train, differentiation, islets, beta cells

## Abstract

**Introduction:** Human pluripotent stem cells (hPSCs) provide many opportunities for application in regenerative medicine due to their ability to differentiate into cells from all three germ layers, proliferate indefinitely, and replace damaged or dysfunctional cells. However, such cell replacement therapies require the economical generation of clinically relevant cell numbers. Whereas culturing hPSCs as a two-dimensional monolayer is widely used and relatively simple to perform, their culture as suspended three-dimensional aggregates may enable more economical production in large-scale stirred tank bioreactors. To be more relevant to this biomanufacturing, bench-scale differentiation studies should be initiated from aggregated hPSC cultures.

**Methods:** We compared five available bench-scale platforms for generating undifferentiated cell aggregates of human embryonic stem cells (hESCs) using AggreWell™ plates, low attachment plates on an orbital shaker, roller bottles, spinner flasks, and vertical-wheel bioreactors (PBS-Minis). Thereafter, we demonstrated the incorporation of an hPSC aggregation step prior to directed differentiation to pancreatic progenitors and endocrine cells.

**Results and discussion:** The AggreWell™ system had the highest aggregation yield. The initial cell concentrations had an impact on the size of aggregates generated when using AggreWell™ plates as well as in roller bottles. However, aggregates made with low attachment plates, spinner flasks and PBS-Minis were similar regardless of the initial cell number. Aggregate morphology was compact and relatively homogenously distributed in all platforms except for the roller bottles. The size of aggregates formed in PBS-Minis was modulated by the agitation rate during the aggregation. In all cell culture platforms, the net growth rate of cells in 3D aggregates was lower (range: −0.01–0.022 h^−1^) than cells growing as a monolayer (range: 0.039–0.045 h^−1^). Overall, this study describes operating ranges that yield high-quality undifferentiated hESC aggregates using several of the most commonly used bench-scale cell culture platforms. In all of these systems, methods were identified to obtain PSC aggregates with greater than 70% viability, and mean diameters between 60 and 260 mm. Finally, we showed the capacity of hPSC aggregates formed with PBS-Minis to differentiate into viable pancreatic progenitors and endocrine cell types.

## Introduction

Human pluripotent stem cells (hPSCs), which include human embryonic stem cells (hESCs) and human induced pluripotent stem cells (hiPSCs), can differentiate into cells from all three germ layers (Thomson, 1998; Takahashi et al., 2007; Yu et al., 2007). In theory, hPSCs can also proliferate indefinitely, making them an attractive starting material for studies on development, disease modelling, drug screening and cell replacement therapies. Stem cell research has revolutionized the field of regenerative medicine by offering potential therapeutic approaches for a range of diseases. For instance, hPSCs can be differentiated into keratinocytes for junctional epidermolysis bullosa (Tamai and Uitto, 2016), retinal pigment epithelial cells for age-related macular degeneration, pancreatic progenitors and endocrine cells for type 1 diabetes, dopaminergic neurons for Parkinson’s disease and numerous blood cell types for bone marrow transplantation and cancer therapy (Blau and Daley, 2019). Rough estimates suggest that ∼1–10 × 10^9^ cells per individual may be needed to replace damaged or dysfunctional cardiomyocytes, hepatocytes, or pancreatic beta cells (Kropp et al., 2017). To reliably generate such clinically relevant cell numbers (hPSCs and their derivatives) there is a need for tight control and characterization of process parameters, cell quality attributes, and raw materials to ensure consistent manufacturing of high-quality hPSCs.

The strategies for expanding hPSCs must be carefully considered. hPSCs may be cultured in static two-dimensional (2D) matrix-coated vessels as monolayers, or in stirred suspension grown on microcarriers that similarly provide a substrate for cell attachment, or grown as three-dimensional (3D) cell aggregates. Furthermore, medium composition as well as feeding strategies can impact the quality and survival of the cells (Kropp et al., 2017).

Cell-to-cell and cell-to-extracellular matrix interactions play important roles in hPSC quality, survival and cell fate (Xu et al., 2001; Braam et al., 2008; Brafman et al., 2013). Initially growing as tightly packed colonies as a monolayer, once hESCs reach ∼80% confluence, they are typically harvested as small clumps and reseeded at a lower cell concentration on a matrix-coated vessel to permit further proliferation. Cell adhesion integrin receptors play an important role in establishing interactions between the extracellular matrix and the cells, and may impact their self-renewal capacity and pluripotency (Braam et al., 2008; Rowland et al., 2009; Vitillo and Kimber, 2017). For instance, Laperle et al. demonstrated that when endogenous α-5 laminin production was disrupted in H9 hESCs and 19-9-11 iPSCs, this resulted in decreased proliferation and increased apoptosis without impacting their pluripotency (Laperle et al., 2015). This phenotype was partially rescued, in a dose-dependent manner, by supplying exogenous laminin 521.

Despite the importance of cell-to-extracellular matrix interactions when grown on a 2D monolayer, the 2D environment does not replicate the complex, 3D organization of tissues. 3D culture systems can better replicate cell-cell interactions, chemical gradients, and mechanical forces found in developing tissues. Moreover, 3D cultures can provide larger surface areas for cell growth, making 3D cultures more amendable to large-scale manufacturing. 3D suspension cultures also offer the potential for more integrated inline monitoring and control of process parameters such as pH and oxygen tension. 3D aggregates can be made with a variety of methods including multi-well plates, stirred tank bioreactors and roller bottles. In particular, roller bottles are currently being employed in the production of hESC-derived pancreatic endoderm cell aggregates for clinical trials (Schulz, 2015). However, several bottlenecks are linked to the formation and expansion of hPSC aggregates. Extreme cell losses can occur during the aggregation process; for example, in one report the aggregate formation efficiency of ∼1% was improved up to 8% with the addition of dextran sulphate (Lipsitz et al., 2018), a large improvement but still a relatively low recovery. While the doubling time of some hESCs can range from ∼20 to ∼56 h (Ware et al., 2006; Panyutin et al., 2017), longer doubling times, slower growth rates and lower fold expansions have been reported for aggregate-based expansion methods compared to monolayer expansion (summarized in Borys et al., 2020). Thus, despite the potential advantages of 3D culture systems, optimizing their culture conditions and addressing bottlenecks remain crucial for their successful application.

Cell aggregates may be formed using an inoculum of single cells (Zweigerdt et al., 2011; Schulz et al., 2012) or cell clumps (Borys et al., 2020). Regardless of how aggregates are generated and cultured, as they grow and increase in diameter, diffusion gradients such as of nutrients, and pH will impact the cell proliferation. Oxygen diffusion limitation can occur as the diameter of hPSC aggregates approach 300 µm (Sart et al., 2017) Modelling studies by Van Winkle and colleagues found oxygen concentration was 50% lower in large hESC clusters (800 µm diameter) compared to smaller clusters (400 µm diameter) (Van Winkle et al., 2012). Heterogeneity in aggregate size can impact cell viability and growth kinetics (Lipsitz et al., 2018; Miranda et al., 2018). Furthermore, undesired morphogen gradients may arise when exposing larger aggregates to differentiation-inducing factors. As such, aggregate size is an important process parameter that can impact downstream applications (Freyer and Sutherland, 1985; Sen et al., 2001; Bauwens et al., 2008; Dahlmann et al., 2013; Kempf et al., 2014; Sart et al., 2017). For instance, Bauwens et al. showed that endogenous levels of extraembryonic endoderm tissue, a parameter that can be modulated by aggregate size, affected the differentiation of hESCs towards cardiomyocyte (Bauwens et al., 2011). Similarly, cardiac differentiation is influenced by the initial size of hESC aggregates (Bauwens et al., 2008). Larger mouse ESC clusters (450 µm diameter) were biased to cardiomyocyte lineage, while endothelial differentiation was enhanced in small clusters (150 µm diameter) (Hwang et al., 2009). Even *in vivo* the importance of cluster size is apparent, though with a need to accommodate much lower blood oxygen levels. For example, islet size across species is conserved with an average of 150 µm in diameter (range: 50–500 µm) (Ionescu-Tirgoviste et al., 2015). *Ex-vivo* human islets are susceptible to size-dependent hypoxia and necrosis in the center of the cluster (Komatsu et al., 2017), underscoring the need to limit size to maximize cell viability. Therefore, controlling aggregate sizes is crucial in bioprocess to minimize the potential of inconsistent morphogen gradients within the clusters that can result in loss of pluripotency, spontaneous or asynchronous differentiation and poor viability. To optimize the downstream differentiation process starting from aggregates of hESCs, there is a need to first develop high performance methods to generate those aggregates.

In this study, we aimed to understand the impact that alternative bench-scale cell culture platforms have on the formation of hESC aggregates. We analyzed the impact of seeding single cells at concentrations from 0.2 to 2 × 10^6^ cells/mL in AggreWell™ plates, low attachment plates on an orbital shaker, roller bottles, spinner flasks and vertical-wheel bioreactors (PBS-Minis). In the case of the PBS-Mini, a range of RPMs during aggregation were investigated. The aggregates were cultured for up to 5 days and characterized for morphology, aggregation yield, growth kinetics, viability, pluripotency and aggregate diameter size distributions. We illustrate the variability between independent replicates and identified aggregation yield as a process parameter that can impact aggregate diameter.

## Materials and methods

### Monolayer cell culture maintenance

H1 hESCs were obtained from WiCell and cultured under feeder-free conditions on 0.27 mg/mL reduced growth factor matrigel-coated (Corning) vessels with mTeSR™1 (STEMCELL Technologies). Cells were passaged using Gentle Cell Dissociation Reagent (GCDR) (STEMCELL Technologies) when confluence was ∼80%. Briefly, the spent media was aspirated, the monolayer was rinsed with PBS without Ca^2+^/Mg^2+^(PBS−/−) (Thermo Fisher Scientific) and incubated with GDRC at 37°C for up to 6 min. GDRC was diluted with fresh mTeSR™1 to stop further cell detachment. The cell suspension was collected and centrifuged for 5 min at 1,000 revolutions per minute (rpm). The supernatant was discarded, and the cells were gently resuspended in mTeSR™1 supplemented with 10 μM Y27632. Duplicate aliquots were collected for cell counts and viability using the Nucleocounter-200^®^ (NC-200™; Chemometec), and the cells were reseeded at the appropriate densities. For a 3-, 4- or 5-day schedule, the seeding densities used were 10,000 cells/cm^2^, 15,000 cells/cm^2^, or 20,000 cells/cm^2^, respectively. Subsequent feeds were done with a complete media exchange using mTeSR™1 only. Cells were cultured under standard conditions: 21% O_2_, 5% CO_2_, 37°C, and fed daily. Following thaw from cryopreservation, the cell cultures did not exceed four passages before re-seeding for aggregation. All experiments with cell lines were approved by the Canadian Stem Cell Oversight Committee and/or UBC Clinical Research Ethics Board.

### Formation of aggregates using different cell culture platforms

**FIGURE 1:**
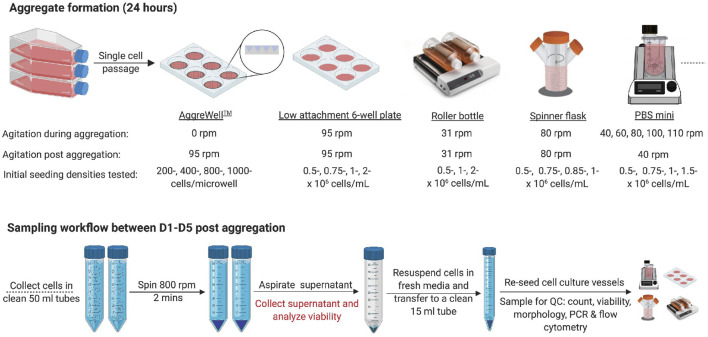
Experimental outline **(A)** Schematic overview of the experimental conditions tested for the aggregate formation and subsequent cell culture using numerous cell culture platforms. D1, D5 = 1 day or 5 days post-aggregation, respectively. Created with BioRender.com.

Once the cells reached ∼80% confluence, they were passaged as single cells with TrypLE™ Express (Invitrogen) using a similar workflow as outlined above. After determining the cell count, cells were reseeded in the respective cell culture platforms at the appropriate seeding density or cell concentration in mTeSR™1 supplemented with 10 μM Y27632 (Figure 1). Unless stated otherwise, 24 ± 2 h later, the media was changed to mTeSR™1, and all experiments were maintained for up to 5 days post-aggregation in mTeSR™1 only.

To determine the effect of the initial seeding density on aggregate formation using AggreWell400™ plates (STEMCELL Technologies), 200-, 400-, 800- or 1,000 cells/microwell were seeded and cultured overnight. Cell aggregates were dislodged by gently triturating each well with a 10 mL serological pipette, collected in 50 mL falcon tubes and spun at 800 rpm for 2 min. The supernatant was discarded or analyzed for cell viability. Recovered clusters were resuspended in fresh mTeSR™1, and duplicate cell counts were done using the NC-200™. Additional cell suspension aliquots were collected for aggregate sizing and viability staining. After aggregation, the cells were transferred to low attachment 6 well plates (Corning) on an orbital incubator shaker (Kuhner, LT-X) at 95 rpm (19 mm throw). Cells were fed with 5 mL/well mTeSR™1 daily.

To determine the impact of the initial seeding concentration on aggregate formation in 6 well plates on an orbital shaker, 0.5–2 × 10^6^ cells/mL were tested in a working volume of 5 mL/well. Complete daily media changes were performed. Wells were visually inspected, pooled into a 50 mL falcon tube, spun, resuspended in fresh media and evenly redistributed back into the wells. All aggregation experiments using 6 well plates or AggreWell400™ were done in parallel.

Aggregates were formed in roller bottles (Corning) using 100 mL working volume on a FlexiRoll (Argos Technologies) rotating at 31 rpm. The impact of initial seeding concentrations and subsequent cell expansion was tested using 0.5-, 1- or 2 × 10^6^ cells/mL. Media exchanges were done by collecting the cell suspension, spinning and resuspending in fresh media.

Aggregates were formed with paddle-based spinner flasks (Corning) using 75 mL working volume on a magnetic base (Chemglass), mixing at 80 rpm. The impact of initial seeding concentrations between 0.5–1 × 10^6^ cells/mL was tested. Daily media changes were done as in described with the roller bottle experiments. Some experiments performed in spinner flasks were performed in parallel using roller bottles.

Finally, the impact of either the initial seeding concentration (0.5–1.5 × 10^6^ cells/mL) or the agitation rate (40–110 rpm) was tested using PBS-Minis (PBS Biotech) with 100 mL working volume. Unless otherwise stated, daily media changes were performed as outlined above. Samples were collected for further downstream analysis. All conditions tested are summarized in Figure 1.

### Sampling, cell count, viability and aggregate size measurement

To evaluate the impact of each experimental condition on the growth kinetics of the cells, daily sampling was done after each complete media exchange. Briefly, the cell suspension was collected and spun for 2 min at 800 rpm. Cell aggregates were resuspended in up to 15 mL of mTeSR™1 and transferred to a 15 mL falcon tube. The suspension was mixed by gently inverting the tube before collecting several 100 µL aliquots for cell counts and viability using the NC-200™. Aggregates were also stained with LIVE/DEAD™ dyes (Thermo Fisher Scientific) using 2 µM calcein-AM and 4 µM ethidium bromide for 30 min in the dark at room temperature (RT). As a control, aggregates were incubated in either 70% ethanol or dimethyl sulfoxide (DMSO) for 15 min before staining with LIVE/DEAD™. Fluorescence images were acquired on an AxioZoom microscope (ZEISS). To assess cell recovery ∼24 h after seeding into a new cell culture vessel, the aggregation yield or plating efficiency was determined using the following equation:

$$\text{Aggregation yield or plating efficiency }\left(\%\right)=N_{1}/N_{0}\;x\;100$$

where N_0_ is the initial total cell count at t = 0 h hours (h), and N_1_ is the total cell count at t = 24 h post-aggregation unless stated otherwise. The aggregation yield quantifies cell recovery following the period of aggregation and can not distinguish contributions of cell growth or loss. A plating efficiency of 100% suggests that all seeded cell clumps reattached to the matrigel-coated surface. The fold expansion was calculated using the following equation:

$$\text{Fold change}=N_{f}/N_{1}$$

where N_1_ and N_f_ are the total cell count at 1 day and the final day post-aggregation, respectively. The net growth rates and the doubling times were determined using the following equation:

$$\text{Net growth rate }\left({µ}_{\text{net}}\right)=\text{In }\left(N_{f}\right)\;–\text{ In }\left(N_{1}\right)/\Delta t$$

$$\text{Doubling time }\left(T_{d}\right)=\text{In }\left(2\right)/{µ}_{\text{net}}$$

Where N_1_ and N_f_ are the total cell count at 1 day and the final day post-aggregation, respectively, and Δt is the time (h) elapsed between day 1 and the final day post-aggregation.

Aggregate morphology was captured daily using a Primovert microscope (ZEISS), and diameter was determined using a semi-automated pipeline in FIJI (ImageJ) after background subtraction using Adobe Photoshop (Adobe Inc.) (Supplementary Figure S1). Between 3-6 independent images were analyzed for a total of 150–1,500 aggregates. The area of each aggregate was determined, and the diameter was calculated using this equation:

$$\text{Diameter }\left(d\right)=2\sqrt{\left(A/\pi \right)}$$

where A is the area of a given aggregate.

### PCR

RNA extraction, reverse transcription qPCR were done as previously described (Schulze et al., 2021). The list of primers is provided in Table 1.

**TABLE 1:** List of primers.

Primer target	Forward	Reverse
SOX2	5'- GAGGAGAGTAAGAAACAGCATGGA -3'	5'- GATTGGTGTTCTCTTTTGCAGC -3'
NFX1	5'-TTTCAGAACAAAGGAGCTTCCAT-3'	5’-TTATCCACACAGCATATCTCATTACA-3'
OCT4	5'- GGGATTAAGTTCTTCATTCACTAAGGAA -3'	5'- CAAGAGCATCATTGAACTTCACCT -3'
FOXA2	5'- ATCGAGGACAAGTGAGAGAGCAA -3'	5'- TGTTATGGATTTCTTCTCCCTTGCG -3'
SOX17	5'- GGTATATTACTGCAACTATCCTGACG -3'	5'- GGAGTCTGAGGATTTCCTTAGCT -3'
CDX2	5'-GAGTTTCACTACAGTCGCTACATCA-3'	5'-GCTGCAACTTCTTCTTGTTGATTTTC -3'
NKX6.1	5'- CCTGTACCCCTCATCAAGGAT -3'	5'- CAAGTATTTTGTTTGTTCGAAAGTCTTC-3'
PDX1	5'- CCCTCTTTTAGTGATACTGGATTGG -3'	5'- CCTTCCAATGTGTATGGTACAGTTTC -3'
NGN3	5'- ACCACCCCATAATCTCATTCAAAG -3'	5'- GTAAGAGACTGAGAGGCAGACAG -3'
NEUROD1	5'- GGTTATGAGACTATCACTGCTCAG -3'	5'- AGAACTGAGACACTCGTCTGTC -3'
PAX4	5'- AGAGGCACTGGAGAAAGAGTTC -3'	5'- CCATTTGGCTCTTCTGTTGGA -3'
ARX	5'- CTCAGCACCACTCAAGACCAA -3'	5'- GCATCCAGACTGCTGTGAAG -3'
INS	5'- GCAGCCTTTGTGAACCAACA -3'	5'- GGTGTGTAGAAGAAGCCTCGTT -3'
GCG	5'- TTCTACAGCACACTACCAGAAGA -3'	5'- CTGGGAAGCTGAGAATGATCTG -3'

### Flow cytometry

Cell monolayers or aggregates were dispersed as single cells using TrypLE™ Express (Invitrogen; Cat# 12604021). Cells were stained for viability with LIVE/DEAD™ fixable aqua dead cell stain (Invitrogen) for 30 min in the dark. Cells were stained with SSEA4 (R&D systems FAF1435, 1:5000), in stain buffer (0.5% BSA, 0.05% sodium azide in PBS) for 30 min prior to fixation in BD Cytofix/Cytoperm™ (BD Biosciences) at RT for 20 min or overnight at 4°C. The cells were washed once with BD perm/wash™ (BD Biosciences) and stained for OCT4 (BD Biosciences 560329, 1:50) and SOX2 (R&D systems IC21018P, 1:1,000) at RT for 45 min. Stage 1 cells (S1D3) were stained for SOX17 (BD Biosciences 561591, 1:500) and FOXA2 (R&D systems IC2400G, 1:100). Flow cytometry was performed using a LSR II flow cytometer (BD Biosciences), and the data was analyzed using FlowJo™ v10 software (BD Life Sciences).

### Dithizone and hypoxia stain

Aggregates were stained with 5 mg/mL Dithizone (DTZ, Sigma 194832). Aggregates were incubated in DTZ for 2 min at RT, and rinsed with PBS−/− until the solution was clear before imaging using an AxioZoom V16. To visualize hypoxia, aggregates were incubated in 5 µM Image-iT™ Green Hypoxia Reagent (Invitrogen I14834) for 3 h at 37°C, 21% O_2_ and 5% CO_2_, rinsed with PBS−/− then imaged.

### Suspension differentiation protocol

Pancreatic progenitors and insulin-producing cells were generated from hPSC aggregates using a modified version of our 7-stage differentiation (Rezania et al., 2014). First, hPSC aggregates were formed from single cells in 0.1 or 0.5 PBS-Minis using 1 × 10^6^ cells/mL, mixing at 60 rpm in mTeSR™1 and 10 µM Y27632. One day after the initial seed, the media was changed to mTeSR™1 and the agitation was reduced to 40 rpm. On day 2, cell counts were determined and reseeded using the following parameters in order to start directed differentiation: 1) hPSC aggregates made in 0.1 Minis were reseeded into 0.1 Mini at 0.5, 0.75 and 1 × 10^6^ cells/mL, 2) hPSC aggregates made in 0.5 Minis were reseeded into 0.5 Mini at 0.5 × 10^6^ cells/mL and in 6WPs at 0.5 and 1 × 10^6^ cells/mL.

### Statistics

Statistical analysis was performed with GraphPad Prism. Data are shown as either an interquartile range with max and min values or mean +/− standard deviation. Pearson’s correlation was used to evaluate the relationship between the initial seeding conditions and either the aggregation yield or the aggregate diameter 1-day post aggregation (D1). A *p*-value < 0.05 was considered statistically significant.

## Results

### Monolayer morphology and growth kinetics

To evaluate the growth kinetics of hESCs grown on a monolayer, we used the H1 hESC line, as it is a widely-used and well-characterized line (Thomson, 1998; Adewumi et al., 2007; Allegrucci and Young, 2007; Tosca et al., 2015). We identified 3 seeding densities that would be confluent and ready to passage at 3-, 4- or 5 days. The cells were maintained for 3 passages. In all cases, cells grew in compact colonies with phase bright borders and a high nucleus-to-cytoplasm ratio (Supplementary Figure S2A). All conditions had a steady increase in cell number by the end of the expansion period with a minimal lag growth phase and viability >80% on most days (Supplementary Figure S2B–D). The cell concentration reached ∼ 1-, 2- or 2.6 × 10^6^ cells/mL by the end of a 3-, 4 or 5-days of culture, respectively. We calculated the plating efficiency to estimate the percentage of cells attached to a matrigel-coated vessel. Plate efficiencies were between 50%–80% (Supplementary Figure S2E). Interestingly, we observed a slight increase in the plating efficiency as the initial seeding density increased. The doubling time was ∼16–18 h with a net growth rate of ∼0.04 h^-1^ regardless of the initial seeding density (Supplementary Figure S2E), similar to previous reports (Panyutin et al., 2017). The fold change in cell expansion was ∼7.5X, ∼22X and ∼44.5X by days 3, 4 and 5, respectively. The lowest initial seeding density resulted in the highest fold change in cell expansion; however, more time (5 vs. 3 or 4 days) was required to reach the appropriate confluence prior to passaging. Although the highest seeding density resulted in the lowest fold expansion, it reached confluence sooner, which could make it a more suitable option for weekend expansion or experiments with a limited time frame. Furthermore, the cell yield using the highest seeding density can be increased by scaling out the cell culture with multiple vessels.

### Aggregation using AggreWell™ plates

**FIGURE 2:**
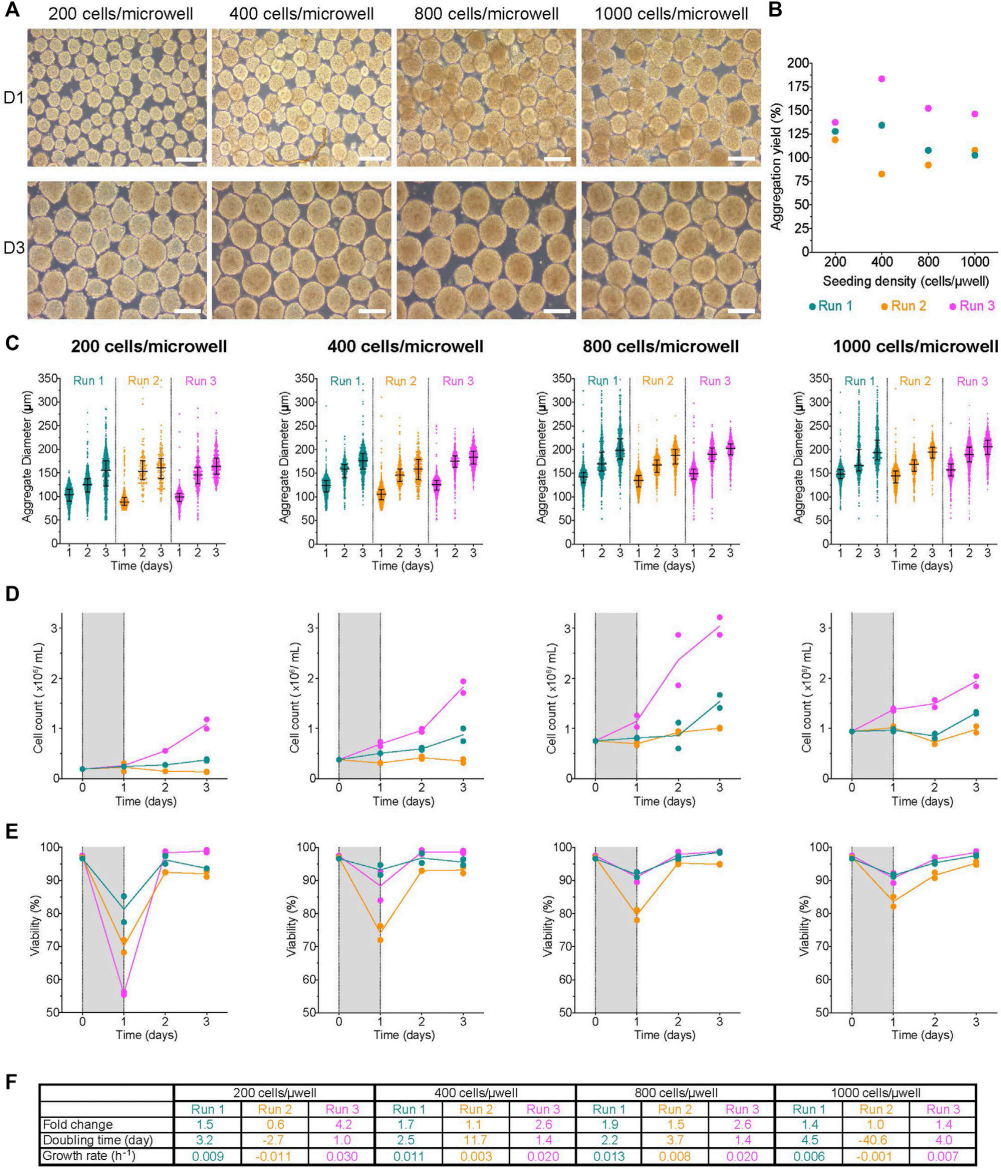
Impact of initial seeding density on aggregate formation in AggreWell™ plates **(A)** Morphology of aggregates at 1- and 3-days post-aggregation, scale bar = 200 µm. **(B)** Aggregation yield for each initial seeding density tested. **(C)** Daily aggregate size distribution displayed as individual diameters with the interquartile range (IQR), **(D)** mean cell concentration, and **(E)** mean viability of aggregates formed after an initial seeding density of 200-, 400-, 800- or 1,000 cells/microwell. **(F)** Table summarizing the fold change, doubling time and growth rate for each run between D1 and D3. The shaded areas in **(D)** and **(E)** indicate the period of aggregate formation, and connecting lines represent the mean of technical duplicates. n = 3 biological replicates/initial seeding density tested. D1, D3 = 1- or 3- days post-aggregation.

AggreWell™ plates can be used to generate and subsequently culture aggregates using a variety of cell types, including hPSCs and their derivatives (Ungrin et al., 2012; van Wilgenburg et al., 2013; Filice et al., 2020; Balboa et al., 2022), mesenchymal stem cells (Baraniak and McDevitt, 2012; Allen et al., 2019), immortalized cell lines (Wrzesinski et al., 2014) and isolated primary cells (Rettinger et al., 2014; Yu et al., 2018). To determine the impact of initial seeding density on undifferentiated H1 aggregate formation in AggreWell™ plates, 200-, 400-, 800- or 1,000 cells/microwell was seeded and statically cultured overnight. Aggregate morphology 1-day post-aggregation (D1) was compact with a smooth periphery in all conditions (Figure 2A, Supplementary Figure S3A). Aggregation yield estimates cell recovery and expansion ∼24 h later and was above 100% of seed quantity in most conditions (Figure 2B). There was a moderate correlation between the initial seed number and the aggregation yield (*r* ^2^ = 0.68, *p*-value = 0.18). However, there was a strong correlation between the initial seed number and the aggregate diameter at D1 (*r* ^2^ = 0.97, *p*-value = 0.01). One day post-aggregation, the aggregate diameter distribution was fairly homogenous between replicate runs (hereafter runs referred to as R1, R2 and R3) of a given condition. The D1 median aggregate diameter generated ranged between 89–104 μm, 106–126 μm, 136–149 μm, and 144–156 µm when 200-, 400-, 800- or 1,000 cells/microwell was seeded, respectively (Supplementary Table S1). We evaluated the D1 viability of the recovered cell clusters and any cells in the supernatant during the media change. The viability of aggregates from all conditions at D1 was high relative to the positive control (Supplementary Figure S3B,C), while the few single cells recovered from the supernatant were non-viable (data not shown).

Aggregates were harvested from the AggreWell™ plates and transferred to low attachment 6-well plates on the Kuhner LT-X orbital shaker for the rest of the experiment. In all conditions, the median aggregate diameter increased daily until day 3 (Figure 2C). By D3, the median cluster diameters had increased by 1.3–1.6X with a range between 155–164 μm, 159–184 μm, 188–203 μm, and 194–207 µm for the 200-, 400-, 800- or 1,000 cells/microwell conditions, respectively (Figure 2C; Supplementary Table S1). Most conditions had a slight increase in cell concentration through the duration of cell culture (Figure 2D); however, we observed higher growth during R3 for all conditions. After an initial drop, the viability in all conditions was maintained at >80% (Figure 2E, Supplementary Figure S3B). Interestingly, the steepest drop in D1 viability was in the 200 cells/microwell condition. There were also variable growth rates, doubling times and fold change expansion between replicate runs (Figure 2F). The fold change in cell expansion between all conditions ranged from 0.6 to 4.2X. The doubling time ranged from 1–4.5 days, with a couple of runs exhibiting population decline. The net growth rate was between 0.003–0.03 h^−1,^ with a few runs having higher death rates based on negative net growth rates. Overall, the growth kinetics of H1 aggregates generated with AggreWell™ plates was slower than H1 cells growing on a 2D monolayer. The data suggest that initial seed and, to an extent, aggregation yield, are process parameters that impact the size of aggregates generated using the AggreWell™ platform. However, the initial seed number had no obvious impact on the measured quality cell attributes (fold change in cell expansion, doubling time and net growth rate).

### Aggregate formation in low attachment 6 well plates on the Kuhner orbital shaker

**FIGURE 3:**
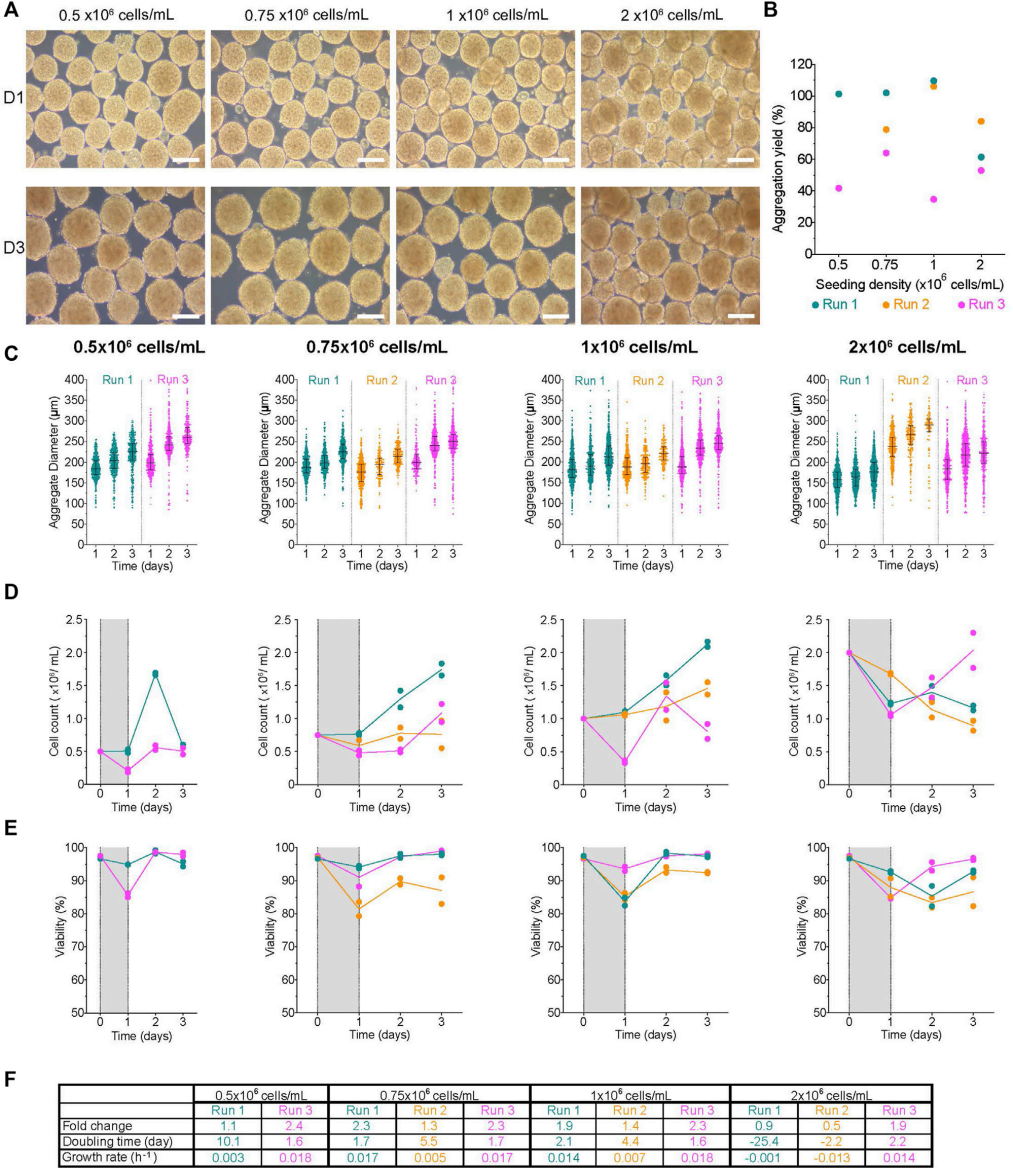
Formation of aggregates in the low attachment 6 well plates on a Kuhner LT-X orbital shaker **(A)** Morphology of aggregates at 1- and 3-days post-aggregation using a Kuhner LT-X at 95 rpm, scale bar = 200 µm. **(B)** Aggregation yield for each initial seeding cell concentration tested. **(C)** Daily aggregate size distribution displayed as individual diameters with the IQR, **(D)** mean cell concentration, and **(E)** mean viability of aggregates formed after an initial seeding cell concentration of 0.5-, 0.75-, 0.85- or 1 × 10^6^ cells/mL. **(F)** Table summarizing the fold change, doubling time and growth rate for each run between D1 and D3. The shaded areas in **(D)** and **(E)** indicate the time of aggregate formation, and connecting lines represent the mean of technical duplicates. n = 2-3 biological replicates/initial seeding cell concentration tested. D1, D3 = 1- or 3- days post-aggregation.

Six well plates are routinely used in labs to generate aggregates for downstream applications like differentiations. Undifferentiated H1 aggregates were generated from single cells in low attachment 6-well plates (6WPs) on the Kuhner LT-X orbital shaker at 95 rpm. The impact of initial seeding concentration (0.5-, 0.75-, 1-, and 2 × 10^6^ cells/mL) on aggregate formation and cell expansion were tested. At D1, the clusters formed from all conditions were compact throughout the experiment (Figure 3A). The aggregation yield was variable between conditions and within replicate runs ranging from ∼38–110% (Figure 3B) and generally lower than observed with AggreWell™ plates despite using the same cell inoculum (Figure 2B). D1 clusters had high viability with minimal viable cells lost to the supernatant (Supplementary Figure S4). There was a weak correlation between the initial cell concentration and either the aggregation yield or the median diameter of aggregates at D1 (vs. aggregation yield: *r* ^2^ = 0.3, *p*-value = 0.45; vs. median diameter: *r* ^2^ = 0.24, *p*-value = 0.5). Interestingly, the range of initial seeding concentrations tested did not have an obvious impact on the size of the aggregates generated at D1 (Figure 3C). At D1, aggregates made from seeding 0.5 × 10^6^ cells/mL had the following median diameters: R1- 184.7 ± 25.9 µm and R3- 198.2 ± 35.7 µm, despite a 2.5X difference in the aggregation yields between the two runs. Day one aggregate size distribution for 1 × 10^6^ cells/mL R1 and R2 were similar to R3 even though there was ∼3.2X difference in aggregation yields between those replicate groups. The median cluster diameter at D1 ranged from 177–199 μm, 181–189 μm, 155–164 μm, and 158–239 µm after an initial seeding concentration of 0.5-, 0.75-, 1-, and 2 × 10^6^ cells/mL, respectively (Figure 3C; Supplementary Table S1). There were variable growth patterns between conditions and replicate runs (Figure 3D). Regardless of growth kinetics, aggregates in all conditions maintained their compact morphology and had an increase in the diameter distribution by the end of the experiment. By D3, the median aggregate diameter was between 226–260 μm, 214–250 μm, 213–246 μm, and 176–290 µm with an initial seeding concentration of 0.5-, 0.75-, 1-, and 2 × 10^6^ cells/mL, respectively. The viability of all conditions was over 80% throughout the experiments (Figure 3E, Supplementary Figure S4). In all conditions, the fold change in cell expansion between D1 and D3 was between 0.5–2.4X (Figure 3F). The 2 × 10^6^ cells/mL condition had the lowest fold change (0.5–1.9X) relative to other conditions, with a decline in cell number in 2 of the 3 replicates (R1 and R2). Overall, the net growth rate, regardless of the initial condition, was between −0.001–0.02 h^−1^, slower than when H1 cells were cultured on a monolayer.

### Aggregate formation in roller bottles

**FIGURE 4:**
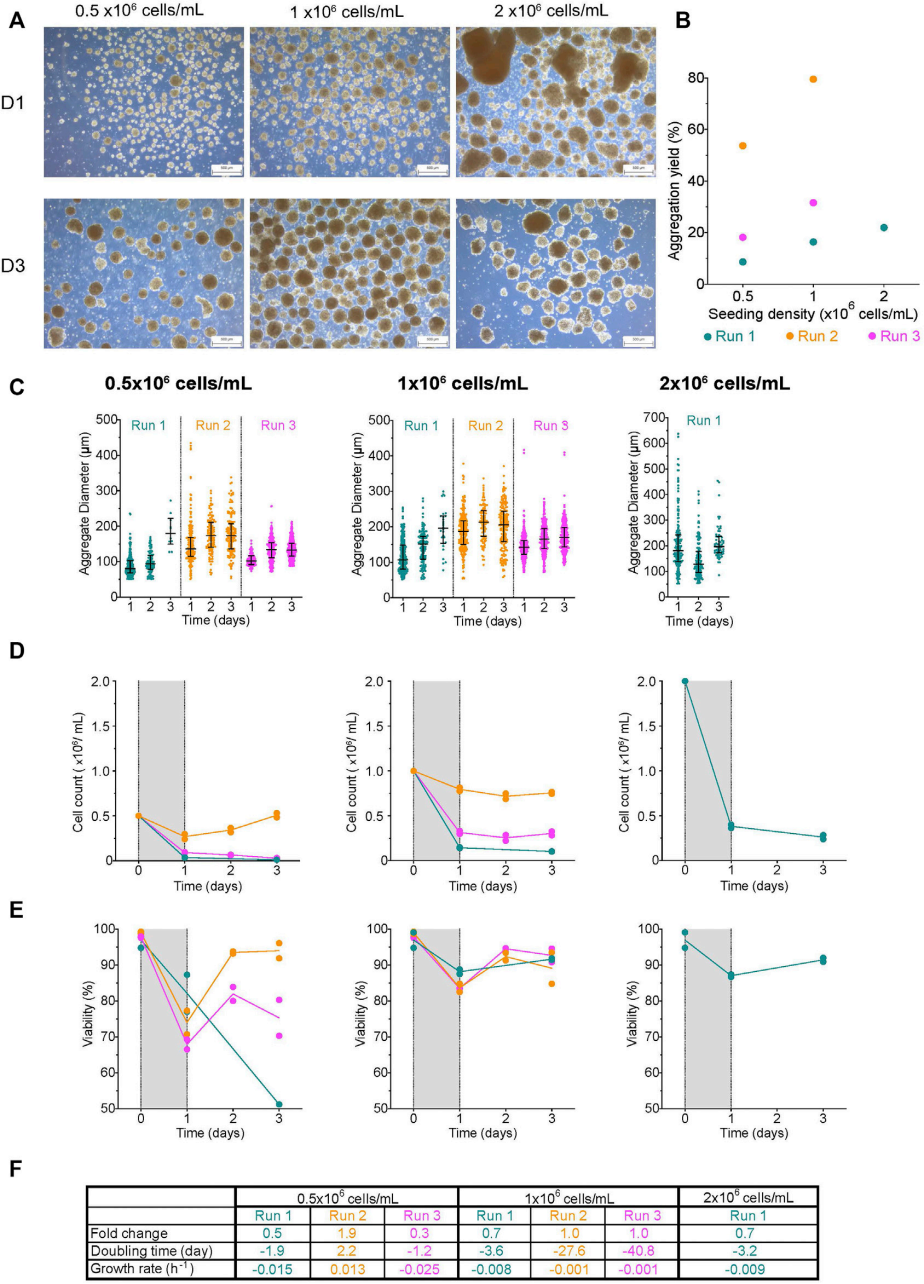
Effect of initial seeding concentration on aggregate formation in roller bottles (**A)** Morphology of aggregates at 1- and 3-days post-aggregation using roller bottler rotating at 31 rpm, scale bar = 500 µm. **(B)** Aggregation yield for each initial seeding cell concentration tested. **(C)** Daily aggregate size distribution displayed as individual diameters with the IQR, **(D)** mean cell concentration, and **(E)** mean viability of aggregates formed after an initial seeding cell concentration of 0.5-, 1-, or 2 × 10^6^ cells/mL. **(F)** Table summarizing the fold change, doubling time and growth rate for each run between D1 and D3. The shaded areas in **(D)** and **(E)** indicate the time of aggregate formation, and connecting lines represent the mean of technical duplicates. n = 1 or 3 biological replicates/initial seeding cell concentration tested. D1, D3 = 1- or 3- days post-aggregation.

**FIGURE 5:**
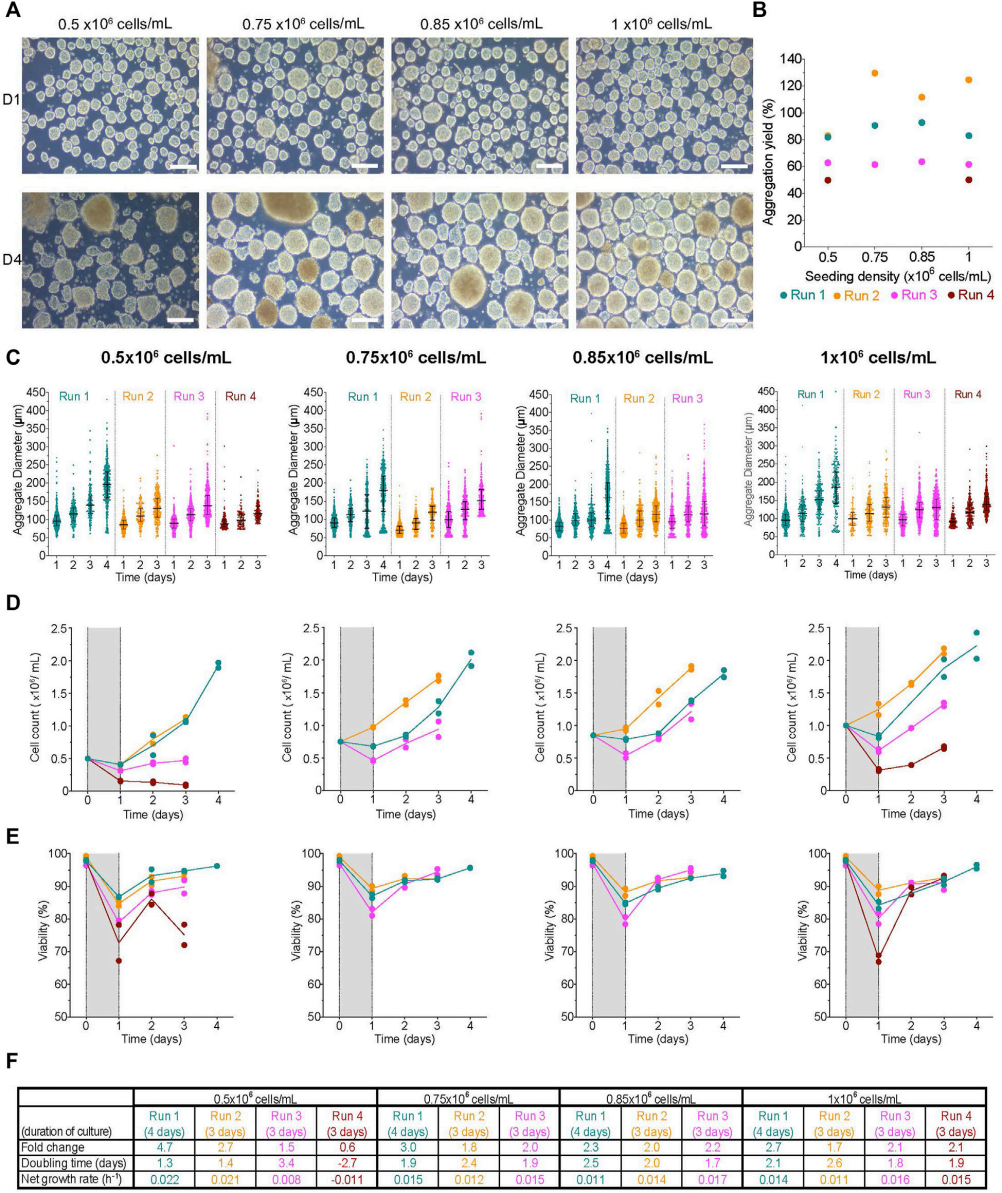
Effect of initial seeding concentration on aggregate formation using spinner flasks **(A)** Morphology of aggregates at 1- and 4-days post-aggregation using spinner flasks mixing at 80 rpm, scale bar = 200 µm. **(B)** Aggregation yield for each initial seeding cell concentration tested. **(C)** Daily aggregate size distribution displayed as individual diameters with the IQR, **(D)** mean cell concentration, and **(E)** mean viability of aggregates formed after an initial seeding cell concentration of 0.5-, 0.75-, 0.85- or 1 × 10^6^ cells/mL. **(F)** Table summarizing the fold change, doubling time and growth rate for each run. The shaded areas in **(D)** and **(E)** indicate the time of aggregate formation, and connecting lines represent the mean of technical duplicates. n = 3-4 biological replicates/initial seeding cell concentration tested. D1, D4 = 1- or 4- days post-aggregation.

Mammalian cells can be cultured in roller bottles as an adherent monolayer (Dhahri et al., 2022), in suspension using microcarriers (Jauregui et al., 2007), or cell aggregates (Schulz et al., 2012; Schulz, 2015). To evaluate how efficiently undifferentiated H1 aggregates could be made in roller bottles, single cells were seeded using an initial cell concentration of 0.5-, 1- or 2 × 10^6^ cells/mL. At D1, aggregates formed with 0.5- and 1 × 10^6^ cells/mL had a compact morphology with a fairly smooth periphery (Figure 4A). Some clusters were more elongated than spherical in shape. The initial seed of 2 × 10^6^ cells/mL resulted in cell sheets and clusters with both tight and loose irregular morphologies. By D3, both 0.5- and 1 × 10^6^ cells/mL had compact aggregates, while the 2 × 10^6^ cells/mL condition had loose aggregates with a jagged periphery. The aggregation yield from most runs was below 40% (Figure 4B). To determine whether the relatively low aggregation yield was due to the death of the remaining cells, we examined the cells in the supernatant at D1. While the viability of clusters from all conditions was high, we found smaller viable clusters as well as viable and non-viable single cells in the supernatant, suggesting that the aggregation yields reported were underestimated (Supplementary Figure S5). Interestingly, as the initial seeding cell concentration increased, so did the aggregation yield and the median diameter of aggregates at D1; however, there was no statistically significant correlation (vs. aggregation yield: r = −0.41, *r* ^2^ = 0.17, *p*-value = 0.73; vs. median diameter: r = 0.98, *r* ^2^ = 0.95, *p*-value = 0.14). The median cluster diameter at D1 had a range from 81–136 μm and 107–187 µm after an initial seeding concentration of 0.5-, and 1 × 10^6^ cells/mL, respectively, while aggregates made with 2 × 10^6^ cells/mL had the broadest range with a median D1 diameter of 181.3 ± 110.5 µm (Figure 4C; Supplementary Table S1). Due to the loss of smaller viable clusters to the supernatant, all reported D1 diameters do not fully represent the range of aggregates generated using roller bottles. Aggregate size increased in most conditions by D3 (median range: 132–180 μm, 170–206 μm, and 197 µm for 0.5-, and 1 × 10^6^ cells/mL conditions, respectively). We observed minimal cell growth in most conditions (Figure 4D). Viability initially dropped at D1 but increased ≥70%, thereafter in most conditions (Figure 4E). Overall, the fold change in most conditions was ≤1, with negative net growth rates indicative of a higher death rate relative to the growth rate (Figure 4F). The lack of cell growth and the subsequent population decline observed is unlikely due to the quality of the cell inoculum as the initial viability was >90%, and the aggregates generated in parallel using spinner flasks showed an increase in cell count (Figure 5).

### Aggregate formation in spinner flasks

To investigate the effects of initial seeding cell concentration on the formation of undifferentiated H1 cell clusters in spinner flasks, we tested 0.5-, 0.75-, 0.85-, or 1 × 10^6^ cells/mL. In all conditions, clusters formed after 1 day of aggregation were relatively even in size, and had smooth boundaries with a compact morphology (Figure 5A). Next, we sought to determine the efficiency of aggregate formation in this bioreactor geometry, as well as any impact the initial cell concentration may have. The aggregation yield was variable between conditions and within their respective replicates with a range between ∼50–130% (Figure 5B). We found the aggregation yield to be most similar between conditions when clusters were formed from the same cell inoculum. For example, the aggregation yield from all conditions during R3 was ∼60%. There was a weak correlation between the initial cell concentration and either the aggregation yield or the median aggregate diameter (vs. aggregation yield: r = 0.47, *r* ^2^ = 0.22, *p*-value = 0.53; vs. median diameter: r = 0.37, *r* ^2^ = 0.14, *p*-value = 0.63). The range of initial cell concentrations tested in this study did not have an obvious impact on the aggregate diameter distribution. The median diameter at D1 for all conditions was between 70–99 µm (Figure 5C; Supplementary Table S1). Clusters had high viability (Supplementary Figure S6A). Next, we tracked the changes in aggregate size over the course of the study. In all conditions, aggregate size increased throughout the experiment (Figure 5C). When extended to 4 days, aggregate size distribution was more heterogeneous. We found there was a deposit of cells on the impeller by the air-liquid interface in all bioreactors regardless of the condition (Supplementary Figure S6B). There were clusters stuck to the bottom of the spinner flasks during every media change (Supplementary Figure S6C). As a result, the diameters of these megaclusters were not measured. In most conditions, there was a steady increase in cell number with minimal time in lag growth phase, and the viability was ≥70% despite the initial drop at D1 (Figures 5D,E). The fold change in cell expansion for 0.5 × 10^6^ cells/mL was between 0.6–4.7X (Figure 5F). For the remaining conditions, the fold change was between 1.7–3. The doubling time, when positive, was between 1.3–3.4 days and the net growth rate was between ∼0.01–0.02 h^−1^. In summary, the range of initial seeding densities had no obvious impact on aggregate made using spinner flasks and or subsequent growth kinetics of the cell clusters.

### Aggregate formation in PBS-Mini bioreactors

PBS-Mini bioreactors can be used to culture several cell types with or without microcarriers (Nogueira et al., 2019; Lembong et al., 2020; Silva et al., 2020; Miranda et al., 2022). We examined the impact of the initial seeding cell concentration and agitation rate on the formation of undifferentiated H1 cell clusters using PBS-Minis. First, we tested the effect of seeding 0.5–1.5 × 10^6^ cells/mL in PBS-Mini bioreactors mixing at 40 rpm. The aggregation yield in all conditions ranged between 49.5%–62.7% with non-viable single cells lost to the supernatant (data not shown). One day post-aggregation, clusters formed in all conditions were compact with a smooth periphery (Supplementary Figure S7A). Aggregates from 0.75–1.5 × 10^6^ cells/mL maintained their compact morphology. By D4, some aggregates generated with 0.5 × 10^6^ cells/mL had irregular periphery with a looser appearance, and cell sheets were present (Supplementary Figure S7A). Interestingly, the initial cell concentrations tested in this study did not modulate aggregate size distribution. The median aggregate diameter from each condition at D1 was as follows: 0.5 × 10^6^ cells/mL was 125.8 ± 25.8 µm, 0.75 × 10^6^ cells/mL was 132.6 ± 28.8 µm, 1 × 10^6^ cells/mL was 143.3 ± 29.3 µm, 1.5 × 10^6^ cells/mL was 147 µm ± 28.8 µm (Supplementary Figure S7B, Supplementary Table S1). Median aggregate size in all conditions steadily increased to between 158–187 µm by D4 (Supplementary Figure S7B). Cell concentration and viability initially dropped at D1 but subsequently increased to up to 1.25 × 10^6^ cells/mL and >80%, respectively, over time in all conditions (Supplementary Figure S7C,D). Overall, growth kinetics were slower than the monolayer control (Supplementary Figure S7E). The doubling time ranged between 2 to 7 days, with a net growth rate between 0.004–0.02 h^−1^. Viability stains also showed some non-viable single cells in the cell suspension at D1 and D5 (Supplementary Figure S7F). All conditions maintained their pluripotency by the end of the expansion period with >96% OCT4+/SOX2+ cells and over 94% SSEA4+ cells (Supplementary Figure S7G). In summary, the initial seeding cell concentrations tested in PBS-Minis did not have an obvious impact on the aggregate size distributions or the aggregation yields. Furthermore, cell expansion and viability were similar between conditions.

**FIGURE 6:**
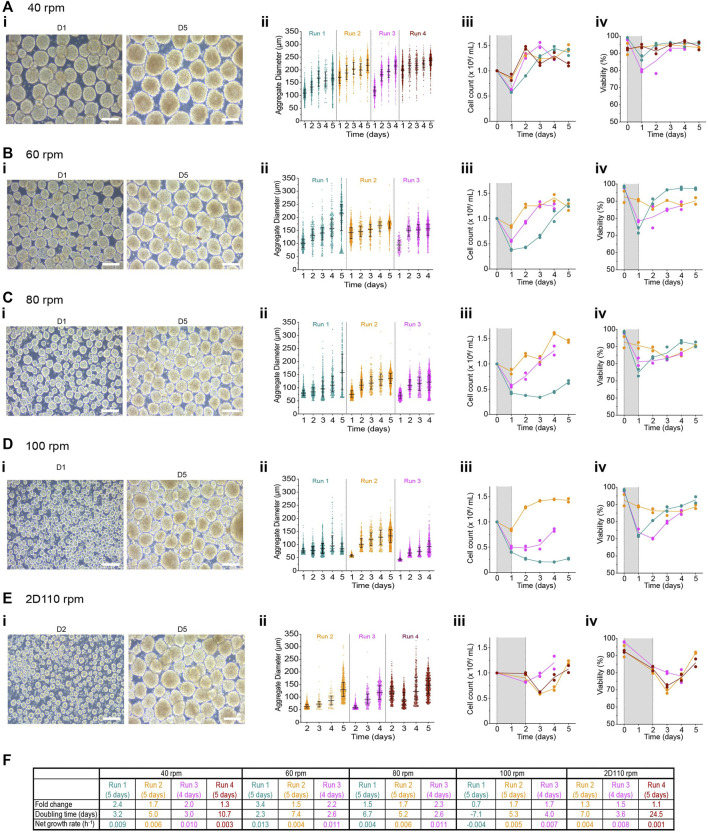
Effect of agitation rate on aggregate formation in PBS-Minis **(i)** Representative morphology of aggregates at 1- and 5-days post-aggregation, **(ii)** daily aggregate size distribution displayed as individual diameters with the IQR, **(iii)** mean cell concentration, and **(iv)** mean viability of aggregates formed using PBS-Mini after an initial seeding cell concentration of 1 × 10^6^ cells/mL mixing at either **(A)** 40-, **(B)** 60-, **(C)** 80 or **(D)** 100 rpm for the first 24 h or **(E)** 110 rpm for the first 48 h (2D110rpm). **(F)** Table summarizing the fold change, doubling time and growth rate for each run. The shaded areas in panels **(iii)** and **(iv)** indicate the time of aggregate formation, and connecting lines represent the mean of technical duplicates. n = 3-4 biological replicates/initial seeding cell concentration tested. Scale bar = 200 μm, D1, D2, D5 = 1-, 2- or 5- days post-aggregation.

To determine the impact of agitation rate on aggregate formation in PBS-Mini bioreactors, 1 × 10^6^ cells/mL were seeded at 40-, 60-, 80-, 100- and 110 rpm for the first 24 h. Aggregates generated from all conditions were tightly packed and fairly symmetric with a smooth periphery. There was a strong inverse correlation between the agitation rate and the D1 diameter (agitation rate vs. D1 diameter: r = −0.98, *r* ^2^ = 0.97, *p*-value = 0.02). D1 aggregate diameter decreased as the agitation rate increased (Figures 6Ai,Bi,Ci,Di,Ei). Within any given run, the aggregation yield was quite similar, regardless of the agitation rate, with a broad range between ∼40–90% (Supplementary Figure S8A). There was a moderate correlation between the agitation rate and the aggregation yield (r = −0.81, *r* ^2^ = 0.65, *p*-value = 0.19). Interestingly, there was some correlation between aggregation yield and the median aggregate diameter at D1 for some agitation rates tested (40 rpm: r = 0.99, *r* ^2^ = 0.97, *p*-value = 0.01; 60 rpm r = 0.86, *r* ^2^ = 0.74, *p*-value = 0.34; 80 rpm r = 0.17, *r* ^2^ = 0.03, *p*-value = 0.9; 100 rpm r = −0.27, *r* ^2^ = 0.07, *p*-value = 0.82). This suggests that aggregation yield may also affect the size of aggregates generated at 40- and 60 rpm.

The D1 aggregates recovered had high viability; however, more non-viable clusters were observed at 110 rpm (Supplementary Figure S8B). Although the supernatant from most conditions had mostly dead cells, viable cells (∼20% of the cell pellet volume) were lost in the supernatant when aggregates were generated at 110 rpm (Supplementary Figure S8B). This loss may be due to a suboptimal centrifugation time preventing the settling of these small-sized aggregates. Viable cell loss was reduced by extending the aggregation period at 110 rpm to 48 h without any media change during that time (referred to as 2D110 rpm). Compared to 110 rpm, aggregates made using 2D110 rpm had a slight dip in cell number by D3 before expanding (Supplementary Figure S8C).

Aggregate diameter increased over time for all conditions (Figures 6Aii, Bii, Cii, Dii, Eii; Supplementary Table S1). Notably, the median D1 diameter between runs varied when generated at 40- or 60 rpm possibly because of the aggregation yields (Figures 6Aii, Bii). The median diameters at D1 were between 109–198 µm for 40 rpm and 94–141 µm for 60 rpm and increased to between 168–236 μm and 172–213 µm respectively by D5. Aggregates generated at either 80- or 100 rpm resulted in fairly consistent diameter distribution between 50–80 μm at D1, and increased to between 133–159 μm and 84–133 µm for 80- and 100 rpm, respectively (Figures 6Cii). Finally, aggregates formed at 110 rpm for 48 h had similar diameters of ∼60 ± 9.4 µm in 2 of 3 runs that increased over the course of expansion (Figure 6Eii).

The growth kinetics between replicate runs was variable, with R1 having the longest lag phases (Figures 6Aiii,Biii,Ciii,Diii,Eiii). Aggregates formed at 40 rpm steadily increased in cell number and viability after the initial dip at D1. Growth kinetics were fairly similar between runs following aggregation at 40 rpm, with a maximum cell concentration at ∼1.5 × 10^6^ cells/mL as early as 2 days post-aggregation (Figure 6Aiii). Viability remained above 70% in both conditions (Figures 6Aiv,Biv,Civ,Div,Eiv). 2D110 rpm had lower viability at day 3 or 4 than other conditions. Based on the parameters characterized above, we further evaluated the pluripotency of the cells at the end of the expansion cycle. Pluripotency was maintained in all conditions. Flow cytometry analysis showed >85% OCT4+/SOX2+ cells and >98% SSEA4+ cells in all conditions at D5 (Supplementary Figure S8D). *OCT4* and *SOX2* expression at the end of the cell culture was similar to or higher than the input cells (Supplementary Figure S8E).

### Integration of aggregation in PBS-Minis before directed differentiation workflow

**FIGURE 7:**
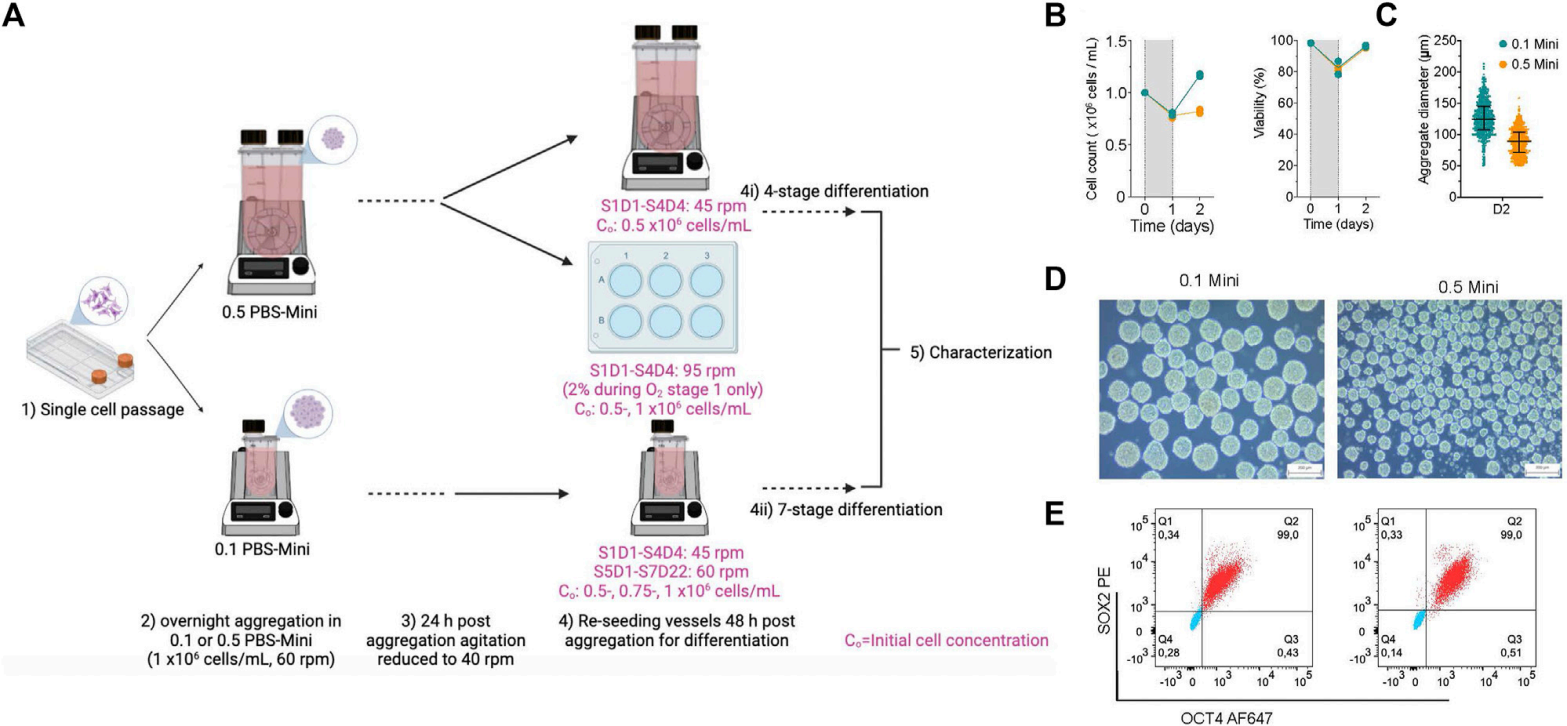
Integration of hPSC aggregation before the start of directed differentiation to pancreatic progenitors and insulin-producing cells **(A)** schematic of experimental design of aggregate formation using 0.1 and 0.5 PBS Minis. Created with BioRender.com. **(B)** Daily cell counts and viability post aggregation. **(C)** Aggregate size distribution, **(D)** morphology and **(E)** flow cytometry of quantification of OCT4 and SOX2 at 2 days (D2) post aggregation and before the stage of differentiation.

To demonstrate the integration of aggregation before initiating differentiation, hPSC clusters made in PBS-Minis were differentiated into pancreatic progenitors and insulin-producing cells (Figure 7A). hPSC aggregates were made using 0.1 and 0.5 PBS-Minis, both mixing at 60 rpm. The viability of aggregates was >90%, and while there was an initial cell loss, cell numbers increased by day 2 in both PBS-Minis, with a more modest increase observed in the 0.5 vessel (Figure 7B). Notably, aggregates made in 0.1 PBS-Minis were larger than those made in 0.5 PBS-Minis (Figures 7C,D). Aggregates from both scales of PBS-Minis had high pluripotency, with 99% SOX2+/OCT4+ cells (Figure 7E). Subsequent differentiations were started 48 h after aggregate formation using 0.1 and 0.5 PBS-Mini, and 6WPs on an orbital shaker.

### Differentiation of aggregates initially formed in 0.5 PBS-Minis to pancreatic progenitor fate

hPSC aggregates generated in 0.5 PBS-Minis were reseeded into a new 0.5 vessel at 0.5 × 10^6^ cells/mL and differentiated to the pancreatic progenitor stage (S4D4). Initial seeding density can impact the formation of pancreatic progenitors (Gage, et al., 2013), therefore we simultaneously seeded 6WPs on an orbital shaker with aggregates at initial concentrations of either 0.5- or 1 × 10^6^ cells/mL. Since cell number can increase during the first stage of differentiation of hPSCs to definitive endoderm (DE) (Rezania et al., 2012), we hypothesized that the aggregates would be susceptible to hypoxia. As a control, aggregates differentiated in 6WPs were cultured under hypoxic conditions (2% O_2_) for the first stage only (S1D1–S1D3). There was little increase in cell number between the initial seed and S1D3 in the 0.5 PBS-Mini condition, unlike both 6WP conditions (Supplementary Figure S9A). Cell numbers steadily declined between S1D3 and S4D4 in the 0.5 PBS-Mini. In contrast, both 6WP conditions increased in cell number up to S2D3 followed by a decline until S4D4. The viability was >90% in most conditions during the differentiation; however, there was a drop to 60% in the 0.5 PBS-Mini condition by S4D4 (Supplementary Figure S9A).

Aggregate morphology and cell fate were evaluated during the 4-stage differentiation. At S1D3, 0.5 PBS-Mini aggregates were less compact and less spherical compared to those in the 6WP (Supplementary Figure S9B). Despite being smaller, there was evidence of hypoxia in S1D3 aggregates from the PBS-Mini, albeit less so than was observed in the 6WP aggregates differentiated at 2% O_2_ (Supplementary Figure S9C). At S1D3, pluripotency markers *OCT4* and *SOX2* were downregulated while DE-related markers *SOX17* and *FOXA2* were upregulated in all conditions, with >90% SOX17+/FOXA2+ cells (Supplementary Figure S9D,E). The data confirmed the successful exit from pluripotency into the DE fate for hPSC aggregate originally generated using 0.5 PBS-Minis.

The remaining stages of differentiation were done at 21% O_2_ for both PBS-Minis and 6WPs. At S2D3, we observed the reorganization of clusters in all conditions (Supplementary Figure S9F). Aggregates differentiating in 0.5 PBS-Minis were predominantly cyst-like with loosely packaged regions compared to the 0.5 × 10^6^ cells/mL 6WP which had more blebs around the periphery, or the 1 × 10^6^ cells/mL 6WP which had a combination of blebs and cystic-like balloons regions. By S3D2, aggregate morphology reverted to being tightly organized in all conditions. By S4D4, aggregates in the 0.5 PBS-Mini condition were smaller compared to those in 6WPs. Expression of *NKX6.1,* and *PDX1* were upregulated in both 6WP conditions confirming the induction of pancreatic progenitor fate (Supplementary Figure S9G). In contrast, 0.5 PBS-Mini aggregates had low *NKX6.1*, *PDX1*, and *NEUROD1* expression and high *CDX2* expression, suggesting an intestinal fate (Coskun et al., 2011).

### Differentiation of aggregates initially formed in 0.1 PBS-Minis to pancreatic insulin-producing cells

**FIGURE 8:**
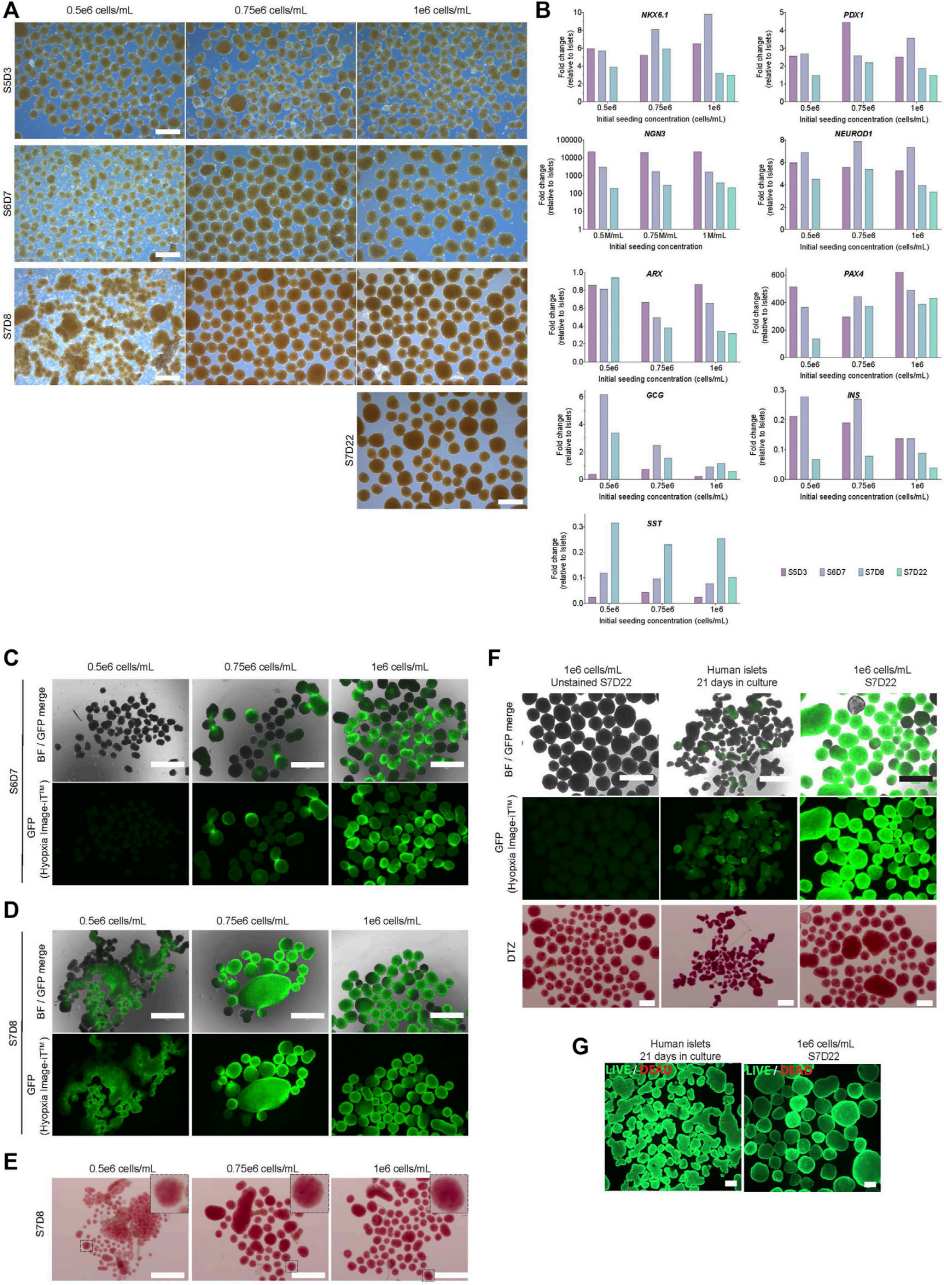
Characterization of cells during seven-stage differentiation using hPSC aggregates generated in a 0.1 PBS mini bioreactor. **(A)** Stage-specific morphology of aggregates initially formed in 0.1 PBS Mini (scale bar = 500 µm). **(B)** Gene expression profile relative to donor human islets **(C)** Hypoxia stain using Hypoxia Image iT™ reagent (green) at S6D7 and **(D)** S7D8. Panels H and I scale bar = 750 µm. **(E)** Dithizone stain at S7D8 **(F)** Hypoxia stain and subsequent dithizone staining of S7D22 clusters and human islets. (Hypoxia scale bar = 750 μm; DTZ scale bar = 500 µm) **(G)** Viability of S7D22 clusters and human islets using calcein-AM (green) and ethidium bromide (red), scale bar = 200 μm. S5D3 = stage 5 days 3, S6D7 = stage 6 days 7, S7D8 = stage 7 days 8, S7D22 = stage 7 days 22.

Based on the generation of pancreatic progenitors (Supplementary Figure S9), we differentiated hPSC aggregates initially formed in 0.1 PBS-Minis to insulin-producing islet-like cells. hPSC aggregates were reseeded in 0.1 PBS-Minis at either 0.5, 0.75 or 1 × 10^6^ cells/mL, differentiated using a 7-stage protocol, and evaluated from the endocrine progenitor stage onwards (stage 5+). Cell number declined in all conditions between stages 5 (S5D3) and 6 (S6D7) with a yield of <1 S6D7 cells/input hPSC (Supplementary Figure S10A,B). Between S5D3 and S7D8+, aggregates from 0.75–1 × 10^6^ cells/mL got bigger while maintaining their spherical shape and viability at >90% (Figure 8A, Supplementary Figure S10C). Aggregates from the 0.5 × 10^6^ cells/mL condition decreased in size and viability, with more single cells observed by S6D7, followed by the formation of pearl-like sheets and megaclusters due to the agglomeration of several clusters at S7D8. The 0.5 and 0.75 × 10^6^ cells/mL seeding conditions were discontinued at S7D8 (Figure 8A, Supplementary Figure S10D).

The predominant endocrine cell types of pancreatic islets include glucagon-producing alpha cells, insulin-producing beta cells and somatostatin-producing delta cells. Expression of *NGN3*, a master regulation of endocrine fate, was upregulated in all conditions, with peak expression at S5D3 followed by a decline (Figure 8B). *NEUROD1*, a downstream target of *NGN3*, was upregulated, with peak expression at S6D7 irrespective of the seeding condition. *PDX1* and *NKX6.1* expression were similar to, if not higher than that measured in human islet controls. Expression of *ARX*, a transcription factor essential for alpha cells, declined in the 0.75 and 1 × 10^6^ cells/mL conditions compared to the 0.5 × 10^6^ cells/mL condition. However, the latter maintained stable expression from S5D3 onwards and was ∼3-fold higher than the other cultures by S7D8 (Figure 8C). Despite similar expression levels between the 0.5- and 1 × 10^6^ cells/mL conditions at S5D3, expression of *PAX4*, a transcription factor important for beta cell fate, decreased ∼4- and 1.5-fold, respectively, between S5D3 and S7D8. There was an increase in *PAX4* expression for the 0.75 × 10^6^ cells/mL condition. At S7D8, *PAX4* expression was similar between 0.75- and 1 × 10^6^ cells/mL condition and 2.7-fold lower in the 0.5 × 10^6^ cells/mL condition. Notably, *PAX4* expression for all conditions was >100-fold higher than human islet controls. *INS* expression peaked by S6D7 for all conditions and declined thereafter. GCG was highest between S6D7 and S7D8 in the cultures seeded at 0.5 × 10^6^ cells/mL, while *SST* expression was similar for all conditions at all timepoint analyzed. Stage 7 was extended to 22 days (S7D22), for the 1 × 10^6^ cells/mL condition only. For most of the genes evaluated at S7D22, their expression levels remained similar to the levels at S7D8, with the exception of the hormone genes, which decreased (Figure 8B).

Given the difference in aggregate size between the conditions, we assessed the clusters for hypoxia from S6D7 onwards. A trend was observed between the initial seeding concentration and the number of positively stained S6D7 aggregates (Figure 8C). Little to no evidence of hypoxia was observed from the 0.5 × 10^6^ cells/mL condition. In contrast, high-intensity hypoxia-positive cells from the other conditions were localized to an arc on the edge of aggregates. By S7D8, hypoxia fluorescence increased in all conditions, with a more even distribution throughout most clusters (Figure 8D). Importantly, despite the presence of hypoxia, there was positive but varying DTZ staining between all conditions (Figure 8E).

Due to the focal pattern of hypoxia dye fluorescence observed at S6D7, we re-stained S7D22 clusters with DTZ after they had been stained and imaged for hypoxia. First, we confirmed that DTZ staining was consistent regardless of prior staining for hypoxia (Figure 8F). Both hypoxia and DTZ stain intensities at S7D22 were higher than those seen at S7D8. Hypoxia fluorescence was higher than human islets cultured for 21 days, and DTZ staining was less intense in the hESC-derivates. The viability of the S7D22 clusters remained high despite the increase in hypoxia intensity during the later stages of differentiation (Figure 8G).

## Discussion

hPSCs are suited for use in regenerative medicine as they can expand indefinitely and differentiate into all cell types. The cell source, passaging technique, feeding schedule, and expansion method used can impact cell yields. With the option of growing cells in a 2D or 3D format, the latter may be better for scale-up production and may more appropriately mimic the native *in vivo* environment, making 3D culture a powerful tool to study and understand human biology more accurately. To make 3D aggregates, we used five commercially available platforms with diverse geometries and scales (AggreWell™, shaking 6-well plates, roller bottles, spinner flasks, and PBS-Mini bioreactors). Minimal processing steps were employed to determine the feasibility of seamlessly incorporating our bioprocess into any workflow. For example, reverse filtration was not used to eliminate remnant single cells immediately after aggregate formation.

**FIGURE 9:**
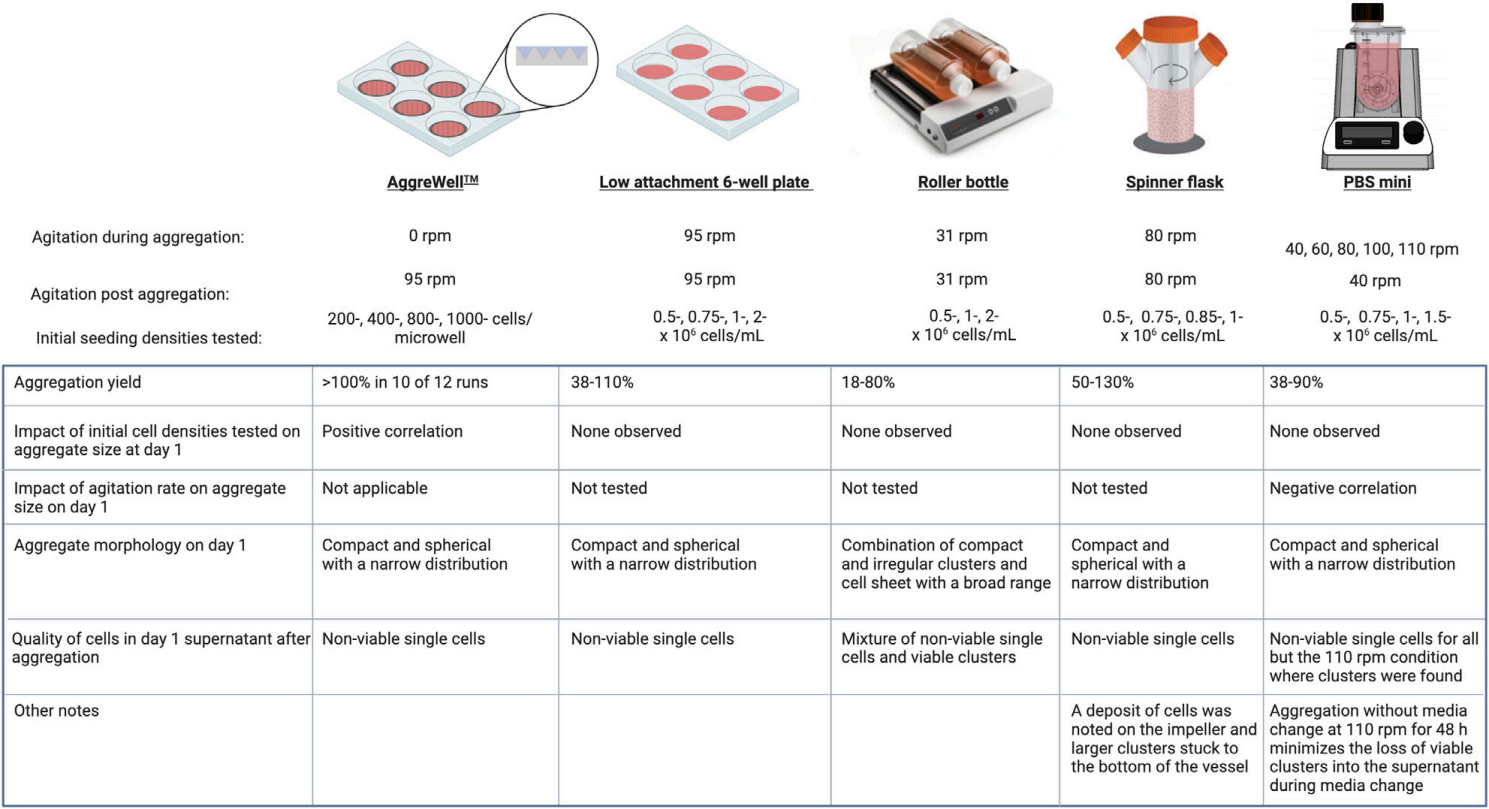
Summary of key findings. Created with BioRender.com.

The cell culture platform design is crucial when culturing stem cells and their derivatives. Ideally, the platform should have monitoring and control strategies for parameters like temperature, provide adequate oxygenation, be scalable, and have a mechanism to facilitate hydrodynamic mixing. Selecting a cell culture platform that generates homogenously sized clusters while minimizing cell loss is not trivial. In this study, we made aggregates using initial seeding concentrations higher (0.25–2 × 10^6^ cells/mL) than those used by others (0.02–1 × 10^6^ cells/mL) (Olmer et al., 2010; Zweigerdt et al., 2011; Abbasalizadeh et al., 2012; Borys et al., 2020; Manstein et al., 2021) with the prospective goal of initiating directed differentiation within 48–72 h post aggregation. We distinguished the impact of the aggregation process on cell recovery (termed aggregation yield) from the subsequent expansion of the remaining cells within aggregates. The AggreWell™ system, which allows high throughput cluster formation, had the highest aggregation yields (≥100% in 10 of 12 runs) of all the platforms tested. Such high efficiency indicates there is cell proliferation during the 24 h aggregation period. Forced confinement of cells in the microwells leads to increased cell-to-cell contact; this provides signaling cues, like e-cadherin interactions, that promote cell survival and self-renewal (Hsiao and Palecek, 2012). There is also minimal shear stress acting on the cells during the static aggregation process in AggreWell™ plates. These features of AggreWell™ plates may create a supportive environment for cell growth and proliferation. In contrast, aggregation yields in the other platforms varied considerably between runs regardless of the initial seeding conditions. The lowest recovery was with the roller bottles, and a trend suggested that the initial seeding concentration may positively correlate to the subsequent aggregation yields. Compared to the AggreWell™ system, these lower cell recoveries from other platforms may be due to differences in the hydrodynamic environment and potential shear stress on the single cells. Indeed, we confirmed that in most instances with low aggregation yields, non-viable single cells were in the supernatant 1 day post-aggregation (Figure 9). Even though attributes of the cell inoculum, such as source, passage number, growth phase and cell viability, may play a crucial role during the aggregation process, we found no trends between the aggregation yields and either passage numbers or the initial viability (>90%).

While we observed high variability in aggregation yield and growth kinetics, precautions were taken to ensure consistent handling during experimentation. This included maintaining uniformity in cell dissociation time, the force of trituration, centrifugation times, cell inoculation, and the duration cells spent outside the incubator. Technical replicate wells (AggreWell™ and 6WPs) were combined for analysis and redistribution, assuming their similarity based on the mass of the aggregates when they were swirled to the center of the plate. We acknowledge that subtle differences while handling the cells could contribute to the observed variability. Furthermore, a more comprehensive analysis of input cell characteristics beyond pluripotency markers, such as nutrient utilization, may inform sources of this variability. We attribute the variability in aggregation yield between replicates to unidentified and uncontrolled process parameters highlighting the importance of control strategies during cell culture processes.

The size and distribution of PSC aggregates is a critical quality attribute that may be controlled by bioprocess parameters such as initial inoculum (single cells, cell clumps or preformed clusters), seeding concentration, agitation rate, encapsulation or addition of surfactants (Ungrin et al., 2008; Lipsitz et al., 2018; Nogueira et al., 2019; Borys et al., 2020; Manstein et al., 2021; Cohen et al., 2023). Because of potential size variations during clump passaging of hESCs which can result in the formation of heterogeneous aggregates, we used single cells instead to seed each cell culture platform to minimize size differences of the input cells between replicates. We found that the initial cell number impacted the size distribution of clusters formed using AggreWell™ plates, similar to other reports (Ungrin et al., 2008), and roller bottles but not the 6-well plates, spinner flask and PBS-Mini bioreactors. The absence of an observable impact of seeding concentration on the aggregate size may be attributed to the range tested or due to insufficient statistical resolution to detect smaller effects. hiPSC aggregates generated with 0.25- and 1 × 10^6^ cells/mL were similar in size, while those made from 2.5 × 10^6^ cells/mL were larger (Lei et al., 2014). There was also a correlation between the initial density and cell expansion over 4 days, regardless of the growth media (mTeSR or E8). In our study, no obvious correlation was found between the initial seeding concentrations tested and subsequent growth kinetics. Lower densities (0.2 and 0.25 × 10^6^ cells/mL), below our evaluated range, can have better fold expansion (4-6X) than higher densities (1X) within 4 days (Abbasalizadeh et al., 2012; Lei et al., 2014). Seeding even lower (0.2 × 10^4^ cells/mL) can result in 11X fold expansion over 5 days (Borys et al., 2020). As seen in the roller bottles, irregular aggregates and a decline in cell number following a 2.5 × 10^6^ cells/mL seed have been reported (Lei et al., 2014), possibly indicating that this density exceeds the capacity for successful aggregation and later proliferation. Hunt *et al.* reported a decrease in the fold change in cell expansion as the initial cell density increased, likely attributed to the build-up of metabolites or reaching a critical aggregate size (Hunt et al., 2014). We demonstrated that aggregate size distribution in PBS-Minis could be controlled by modulating the initial agitation rate similar to others (Borys et al., 2020). Generating aggregates within the first 24 h at 110 rpm in PBS-Minis resulted in small aggregates, some of which may be unintentionally discarded in the supernatant during media changes without sufficient centrifugation or gravity settling time. However, considering that prolonged centrifugation (Archibald et al., 2016) and gravity settling (Lee et al., 2022) can negatively impact cell yield and viability, we chose to extend the aggregation period from 24 h to 48 h. This adjustment resulted in minimal viable cell loss in the supernatant. Finally, we observed that at a given agitation rate, higher aggregation yields could further impact cluster size resulting in larger clusters. Given the variability in observed, identifying the causative parameters could facilitate batch-to-batch reproducibility of the clusters generated.

It has been suggested that hPSCs are sensitive to shear stress resulting from hydrodynamic mixing (Kropp et al., 2017). For impeller-driven bioreactors, the shape and orientation of the impeller can influence the initial aggregation (Yirme et al., 2008). The bioreactor impeller and agitation should allow cell mixing with minimal settling to prevent the fusion of aggregates while maintaining viability and cell identity. Unlike conventional spinner flasks with a horizontal impeller, the PBS-Mini has a U-shaped bottom with a vertical wheel that promotes uniform mixing while operating at a relatively low energy dissipation rate (an alternative measure to shear stress) (Borys et al., 2021; Dang et al., 2021). Aggregates generated using PBS-Minis had a narrow size distribution and remained compact throughout the experiment. Similarly, aggregates made using AggreWell™ plates, 6-well plates, and spinner flasks were compact. Unlike the densely packed aggregates made in our study, Cohen et al. reported the generation of self-organizing cystic-like hPSC aggregates, reminiscent of the epiblast, using a microfluidic encapsulation platform (Cohen et al., 2023). While the implications of this morphology in biomanufacturing are yet to be determined, these lumenized aggregates had double the cell yield compared to 2D cultures and maintained pluripotency and high viability. In our study, cell viability was >70% for all platforms tested. Of note, high viability was maintained for all agitation rates tested in PBS-Minis even when beyond the recommended maximum agitation of 100 rpm. The non-viable fraction, mostly comprised of single cells, was depleted over time during daily media change. Furthermore, the pluripotency of cells in the PBS-Mini was maintained by the end of the experiment (D5). Although recovered clusters were homogenously sized in spinner flasks, agglomerated clusters were stuck at the bottom of the vessel and cells were deposited on the spinner flask impeller at the air-liquid interface. Without an impeller, the clusters formed with 6-well plates were homogenous, whereas those made with roller bottles were not.

Aggregate size and distribution are critical process parameters that can impact cell fate, growth kinetics, cell viability and survival. After the initial drop in viability during the first 24 h of aggregation, cell viability was maintained >70% in all conditions, regardless of the platform used. Except for the roller bottles, all other platforms maintained a narrow size distribution throughout. The mean aggregate diameter obtained from all platforms by day one was between ∼60–260 μm, which falls below the threshold (>300 µm) where diffusion limitation becomes a concern (Sen et al., 2001; Sart et al., 2017). For all platforms evaluated, aggregates had slower net growth rates (range: −0.01–0.022 h^−1^) than cells grown on a 2D monolayer (range: 0.039–0.045 h^−1^). 3D aggregate growth rate may be improved by culturing cells in a perfusion bioreactor system (Manstein et al., 2021), using encapsulation (Lei et al., 2014; Cohen et al., 2023) or using media better suited to support 3D growth (Nogueira et al., 2019). Heterogenous aggregate size distribution can impact growth kinetics. Nath and colleagues showed that small and large hiPSC aggregates (2 × 10^1^ cells/aggregate and 1.3 × 10^3^ cells/aggregate, respectively) had slower growth rates than medium clusters (2.8 × 10^2^ cells/aggregate) (Nath et al., 2017). Eventually, there was low Ki67 immunoreactivity in the center and a collagen type 1 shell around the clusters and minimal proliferation (Nath et al., 2017). All platforms, excluding the roller bottle, had a steady but variable increase in cell number over time and between replicates. A combination of cell growth, death and fusion can impact the final size and morphology of aggregates following extended culture. In hiPSC aggregates, the extrusion of apoptotic cells due to the contraction of neighbouring cells during the growth phase, in addition to forming a collagen type 1 shell over the aggregate, impacted both the compactness and growth of clusters (Kim et al., 2018). The extracellular matrix (ECM) provides structural support and is important in biochemical signal transductions (Chen et al., 2007). Over time, ECM shells can form around H9 aggregates resulting in enhanced diffusion limitation and spontaneous differentiation (Sachlos and Auguste, 2008). In summary, the size and distribution of aggregates play a crucial role in determining cell fate, differentiation, growth kinetics and overall viability.

Pancreatic progenitors and insulin-producing cells have been generated from hPSC aggregates made in 6WPs (Schulz et al., 2012), roller bottles (Schulz, 2015), and spinner flasks (Velazco-Cruz et al., 2019). Here, we evaluated two scales and leveraged the differences in the vertical wheel size of 0.1 and 0.5 PBS-Minis to control aggregate size (Inner diameter: 0.1 PBS-Mini = 3.6 cm**,** 0.5 PBS-Mini = 7.2 cm). Due to the larger wheel diameter in 0.5 PBS-Minis, agitating at 60 rpm generates greater maximum shear at the outer radius of the wheel, compared to 0.1 PBS-Minis. Consequently, smaller aggregates (88 µm mean diameter) were formed in 0.5 PBS-Minis than those made in 0.1 PBS-Minis (126 µm mean diameter) before the start of definitive endoderm differentiation (stage 1).

While the smaller aggregates differentiating in 0.5 PBS-Minis failed to form *NKX6.1* and *PDX1* expressing cells, clusters made from the same inoculum and seeded in 6WPs for differentiation became pancreatic progenitors. Furthermore, by S4D4, there was lower viability in clusters differentiated in 0.5 PBS-Minis (60%) compared to those in the 6WP (97%), perhaps as a result of the greater shear stresses acting on smaller aggregates in the former condition. While we cannot definitively state the optimal size range for hPSC aggregates to generate pancreatic progenitors and insulin-producing cell clusters based on this study, aggregate size can influence several cell quality attributes. Heterogenous aggregate size can impact the diffusion of soluble factors leading to asynchronous expansion and differentiation as well as compromised survival. Therefore, generating hPSC clusters with a narrow distribution should be a goal. We showed that further differentiation of pancreatic progenitors generated endocrine progenitors based on *NGN3* and *NEUROD1* expression. The low cell yield by the S6D7 suggests that further optimization is required for this 3D differentiation protocol. Nevertheless, the intense DTZ stain as well as insulin, glucagon, and somatostatin expression, confirmed the successful generation of viable insulin-producing endocrine cells from hPSC aggregates initially made with PBS-Minis.

## Conclusion

Establishing an optimized seed train is crucial for the successful transition from 2D to 3D cell culture as it enables the consistent and controlled propagation of cells, resulting in the production of a uniform population. Here, we report the formation and expansion of hESC clusters using five different cell culture platforms. Static aggregation using AggreWell™ plates was efficient, with minimal cell loss. In contrast, dynamic aggregation using 6-well plates, spinner flasks, and PBS-Minis resulted in lower aggregation yields, but like the AggreWell™ plates, all three platforms made aggregates with tight distribution and compact morphology. Aggregates generated with roller bottles were looser and heterogeneous in size. Finally, aggregates formed in PBS-Minis at 110 rpm for 48 h had similar diameters of ∼60 ± 9.4 µm in 2 of 3 runs that increased over the course of expansion (Figure 8E). While this study primarily focused on controlling size during aggregate formation and cell recovery over a short time before initiating directed differentiation, cell yields could be improved. Strategies like lower initial seeding concentration, feeding strategy and media formulation can increase cell yield (Nogueira et al., 2019; Borys et al., 2020; Manstein et al., 2021). The results of our study demonstrate the importance of developing an optimized bioprocess workflow which starts by identifying process parameters and cell attributes, stratifying them using a risk-based approach such as quality-by-design to help prioritize critical components, and establishing an acceptable operating range that can be incorporated in an optimized cell culture seed train.

## Data Availability

The original contributions presented in the study are included in the article/Supplementary Material, further inquiries can be directed to the corresponding author.

## Supplementary material

Supplementary Table s1 Summary of the size distribution of aggregate made using AggreWell™ plates, using 6-well plates on an orbital shaker, roller bottles, spinner flasks and PBS-Minis. R, run; IQR, Interquartile range; 6WP, 6-well plates; AG, AggreWell™; SF, spinner flask; RB, roller bottle.

Supplementary Figure S1
**(A)** An illustration of the workflow used to determine aggregate diameter. * indicates macro-based measurement of aggregate area. **(B)** Example of macro run using FIJI for area measurement.

Supplementary Figure S2 Morphology and growth kinetics of adherent H1 cells. **(A)** Daily morphology, **(B)** cell density, **(C)** cell concentration, and **(D)** viability of cells after an initial seeding density of 0.1-, 0.15 or 2 × 10^5^ cells/cm^2^. Panels (**(B–D)**: connecting lines represent the mean of technical duplicates. **(E)** Table summarizing the fold change, doubling time and growth rate for each run. *n* = 2 biological replicates/initial seeding density.

Supplementary Figure S3 Morphology of aggregate before harvesting from the AggreWell™ plates and the viability staining for aggregates generated. **(A)** Aggregate morphology in AggreWellTM plates before harvest. **(B)** Viability staining of clusters 1 day and 3 days post-aggregation (D1 and D3, respectively). **(C)** Viability stain of positive controls pretreated with 70% ethanol or DMSO. All scale bar = 500 µm; green = calcein-AM; red = ethidium bromide.

Supplementary Figure S4 Viability stain of the aggregates generated in 6 well plates on the Kuhner LT-X orbital shaker. **(A)** Viability staining of clusters 1 day and 3 days post-aggregation (D1 and D3, respectively). Scale bars = 500 µm; green = calcein-AM; red = ethidium bromide.

Supplementary Figure S5 Viability stain of the aggregates from roller bottles. **(A)** Viability stains 1-day post-aggregation (D1) on recovered clusters/cell suspension or the supernatant, scale bar = 500 µm; green = calcein-AM; red = ethidium bromide.

Supplementary Figure S6 Viability stain of the aggregates from spinner flasks. **(A)** Viability staining of clusters 1 day and 4 days post-aggregation (D1 and D4 respectively), scale bar = 500 µm ; green = calcein-AM; red = ethidium bromide. **(B)** Representative image of cells deposited on the air-liquid interface of the impeller (black ROI), and **(C)** representative images of cells before media change showing agglomerated clusters that stick to the bottom of the spinner flask (arrows highlight the range in size of clusters that stick).

Supplementary Figure S7 Impact of initial seeding cell concentration on aggregate formation in PBS-Minis. **(A)** Representative morphology of aggregates at 1- and 4-days post-aggregation (D1 and D4, respectively) using PBS-Mini mixing at 40 rpm, scale bar = 200 µm. **(B)** Daily aggregate size distribution displayed as individual diameters with the IQR, **(C)** mean cell concentration, and **(D)** mean viability of aggregates formed after an initial seeding cell concentration of 0.5-, 0.75-, 1- or 1.5 × 106 cells/mL. **(E)** Table summarizing the fold change, doubling time and growth rate for each run **(F)** Viability staining of clusters 1 day and 4 days post-aggregation, scale bar = 500 µm **(G)** flow cytometry data of pluripotency markers SOX2, OCT4 and SSEA4. Blank PE shows the fluorescent in an empty PE channel. The shaded areas in **(C)** and **(D)** indicate the time of aggregate formation.

Supplementary Figure S8 Aggregation yield, viability and pluripotency of aggregates made in PBS-Minis. **(A)** Aggregation yield for each initial agitation rate. **(B)** Viability stains 1-day post-aggregation on recovery clusters or the supernatant, scale bar = 500 µm. **(C)** Cell count and viability of aggregates formed at 110 rpm. **(D)** Representative flow cytometry of pluripotency markers SOX2, OCT4 and SSEA4 (*n* = 3–4 biological replicates/condition). Blank PE shows the fluorescent in an empty PE channel. **(E)** Gene expression data for SOX2 and OCT4 (*n* = 4–5 biological replicates/condition). 2D110RPM = 48 h aggregation at 110 rpm.

Supplementary Figure S9 Characterization of cells during four-stage differentiation using hPSC aggregates generated in a 0.5 PBS mini bioreactor. **(A)** Cell count and viability during four-stage differentiation cultured in 0.5 PBS-Mini or 6WP. **(B)** Morphology of S1D3 aggregates cultured in 0.5 PBS-Mini (21% O_2_) or 6WP (2% O_2_). Scale bar = 500 µm. **(C)** Hypoxia staining of clusters at S1D3 using hPSC aggregates generated in a 0.5 PBS mini bioreactor. Scale bar = 150 µm. **(D)** Gene expression relative to hESCs and **(E)** flow cytometry quantification of indicated markers at S1D3. **(F)** Morphology of S2D3-S4D4 aggregates differentiated in 0.5 PBS-Mini or 6WP. **(G)** Gene expression relative to human islets at S4D4. Scale bar = 500 µm. S1D3 = stage 1 day 3, S2D3 = stage 2 day 3, S3D2 = stage 3 day 2, S4D4 = stage 4 day 4.

Supplementary Figure S10 Cell count and viability during endocrine differentiation. **(A)** Stage-specific cell count. **(B)** S6D7 cell yield. **(C)** Stage-specific viability. **(D)** S7D8 viability. S5D3 = stage 5 day 3, S6D7 = stage 6 day 7, S7D8 = stage 7 day 8.
