# Supplementary figures and images for: Analysis of the effects of bench-scale cell culture platforms and inoculum cell concentrations on PSC aggregate formation and culture
Source: Front Bioeng Biotechnol. 2023 Dec 1;11:1267007. doi: 10.3389/fbioe.2023.1267007 (PMC10722899; doi:10.3389/fbioe.2023.1267007)

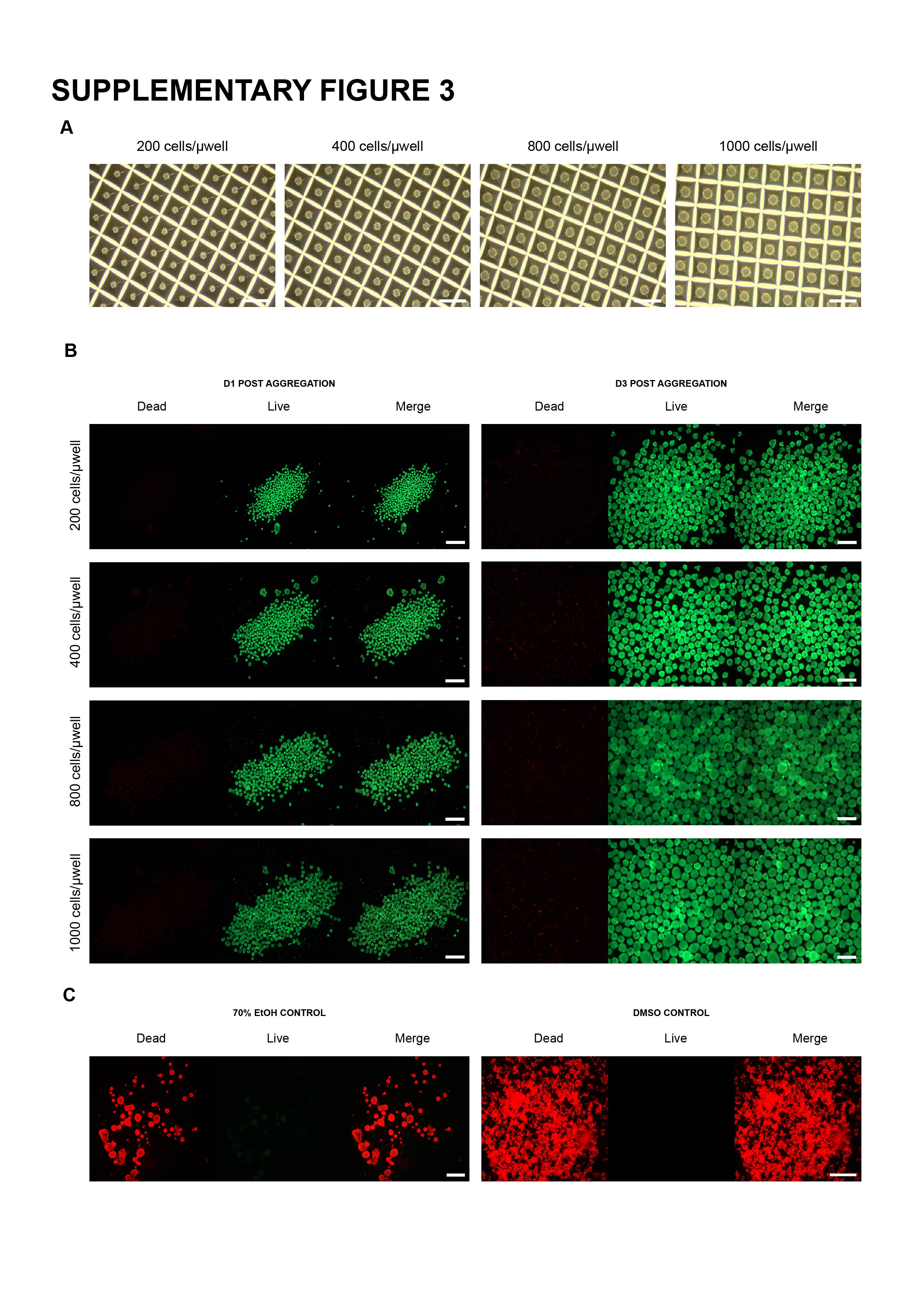

Supplement: Supplementary file 1 [file Image3.JPEG]

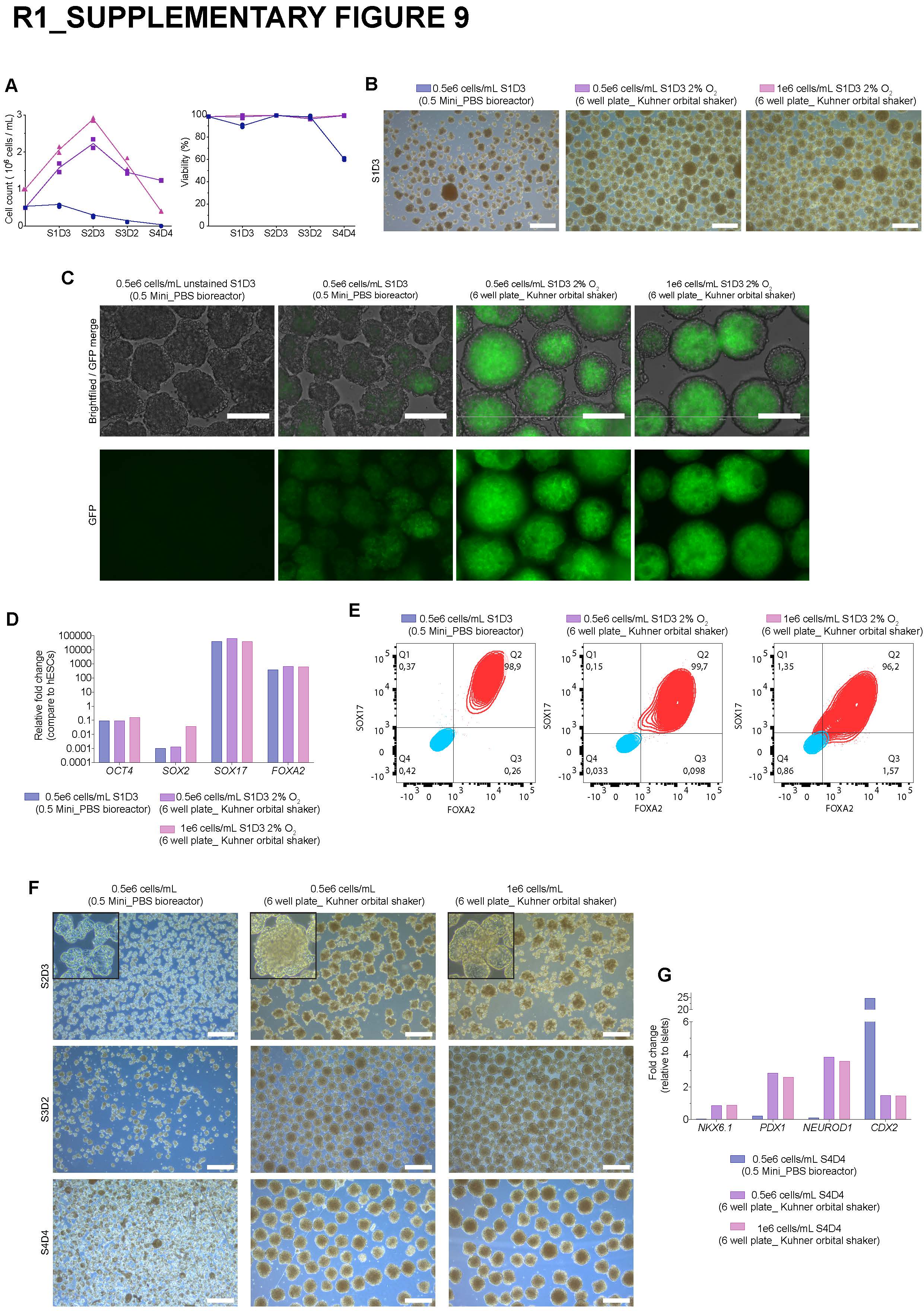

Supplement: Supplementary file 2 [file Image9.JPEG]

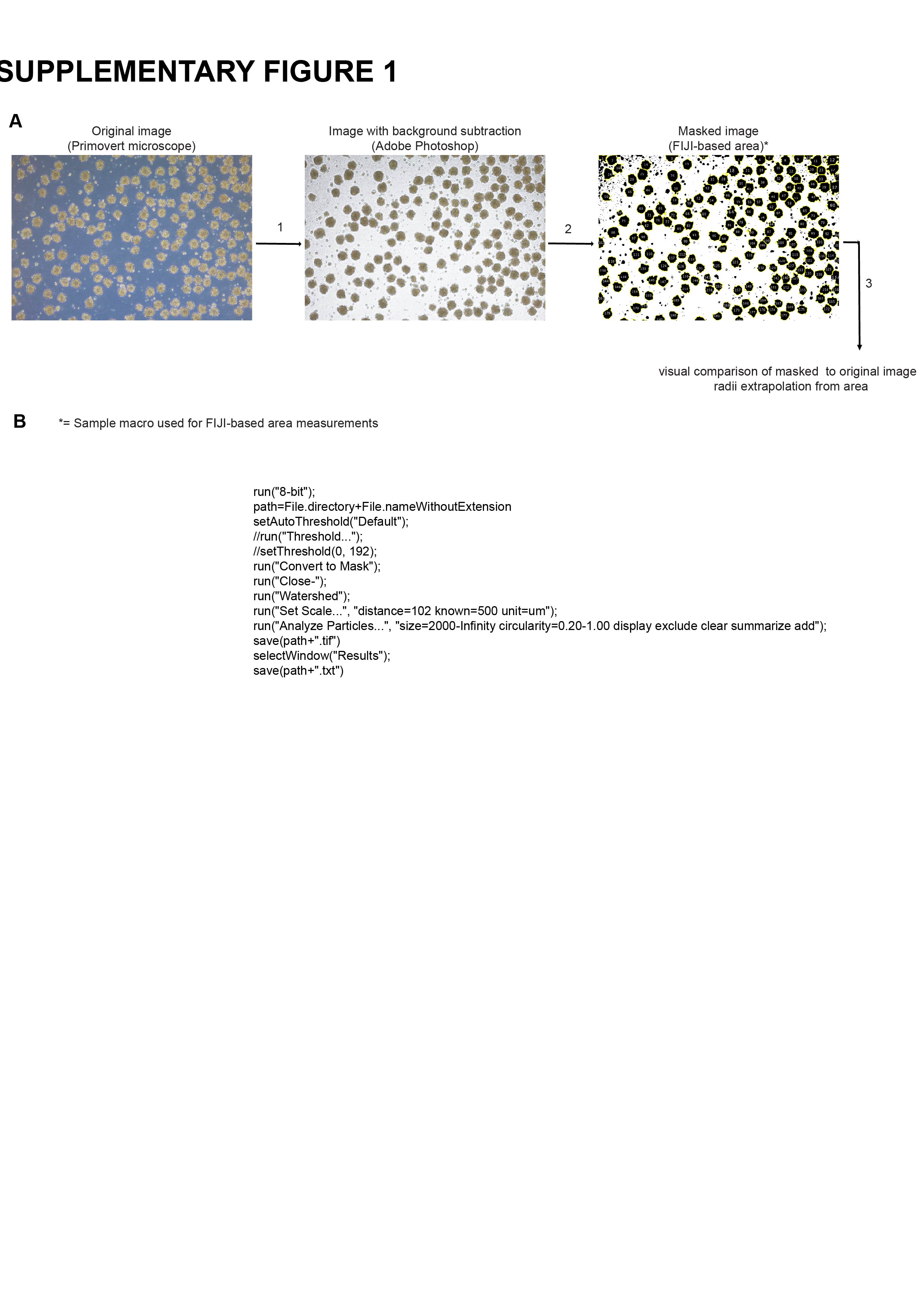

Supplement: Supplementary file 3 [file Image1.JPEG]

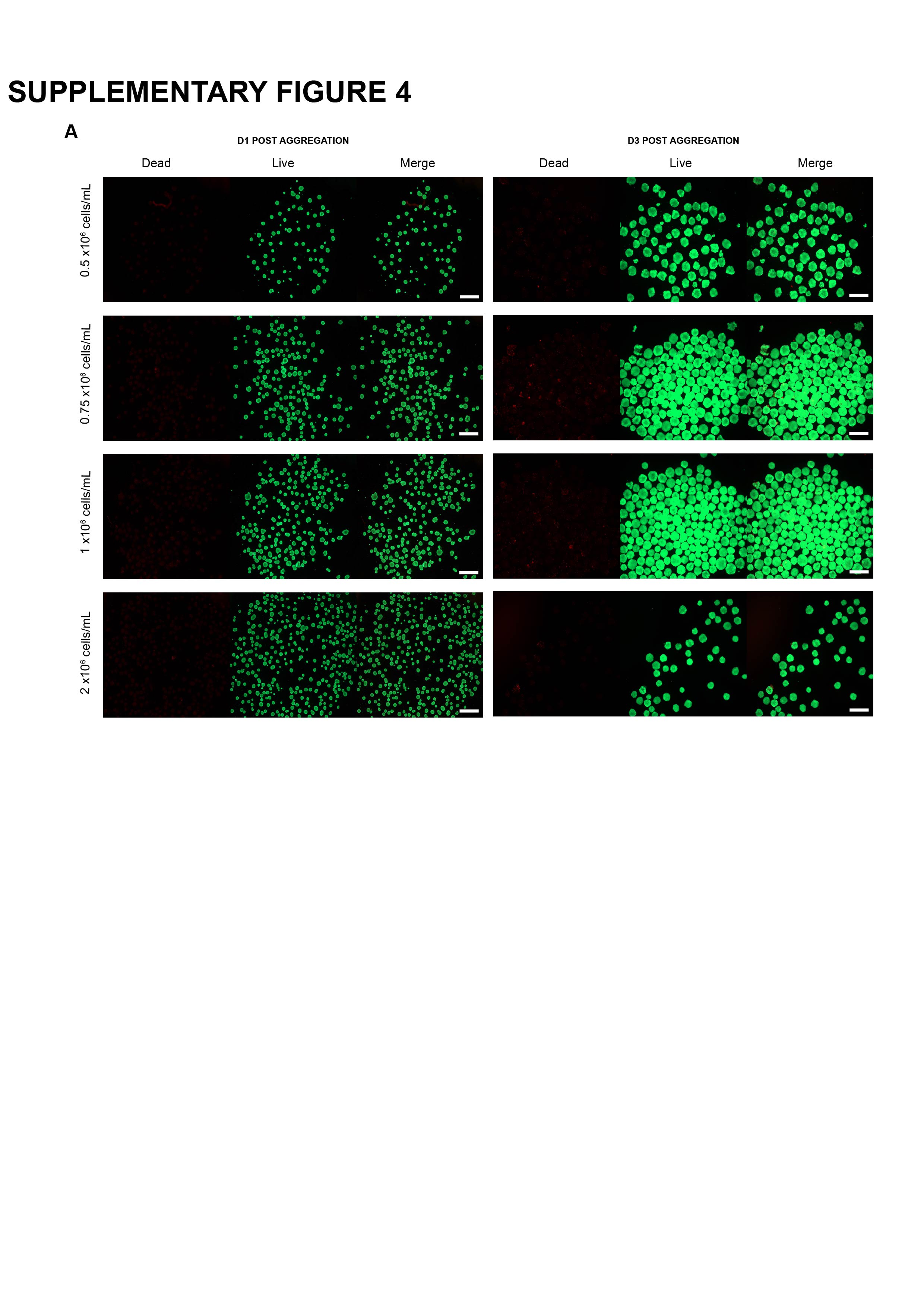

Supplement: Supplementary file 4 [file Image4.JPEG]

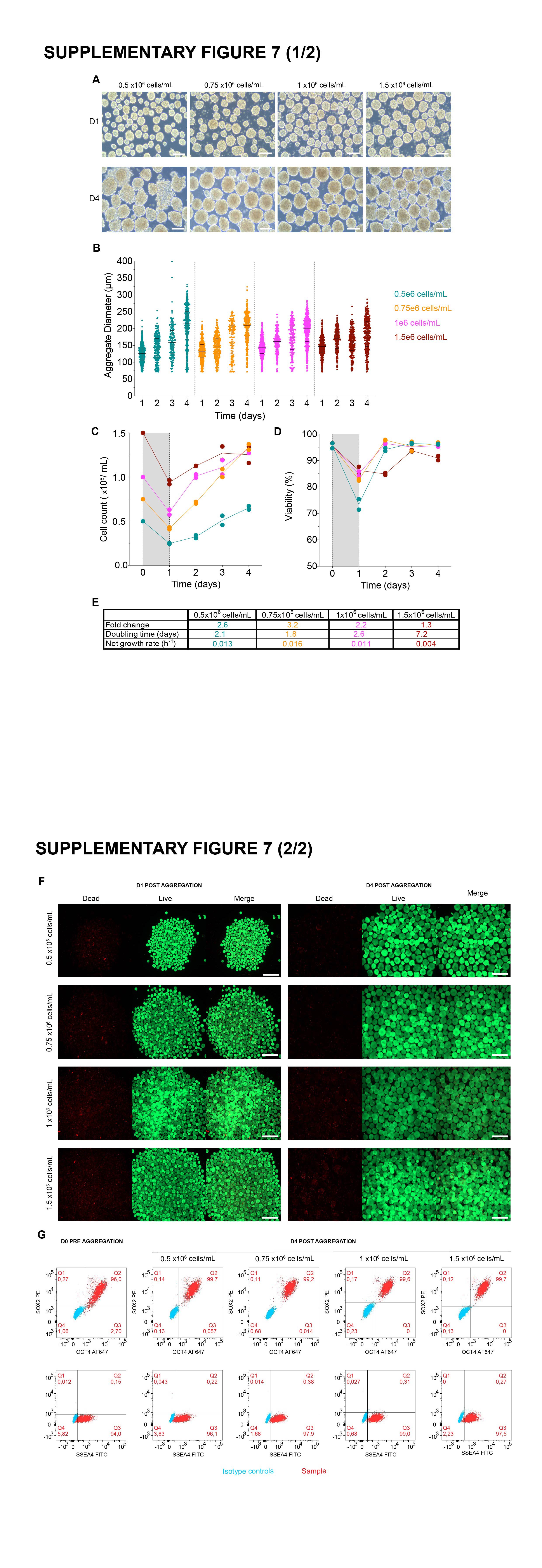

Supplement: Supplementary file 5 [file Image7.JPEG]

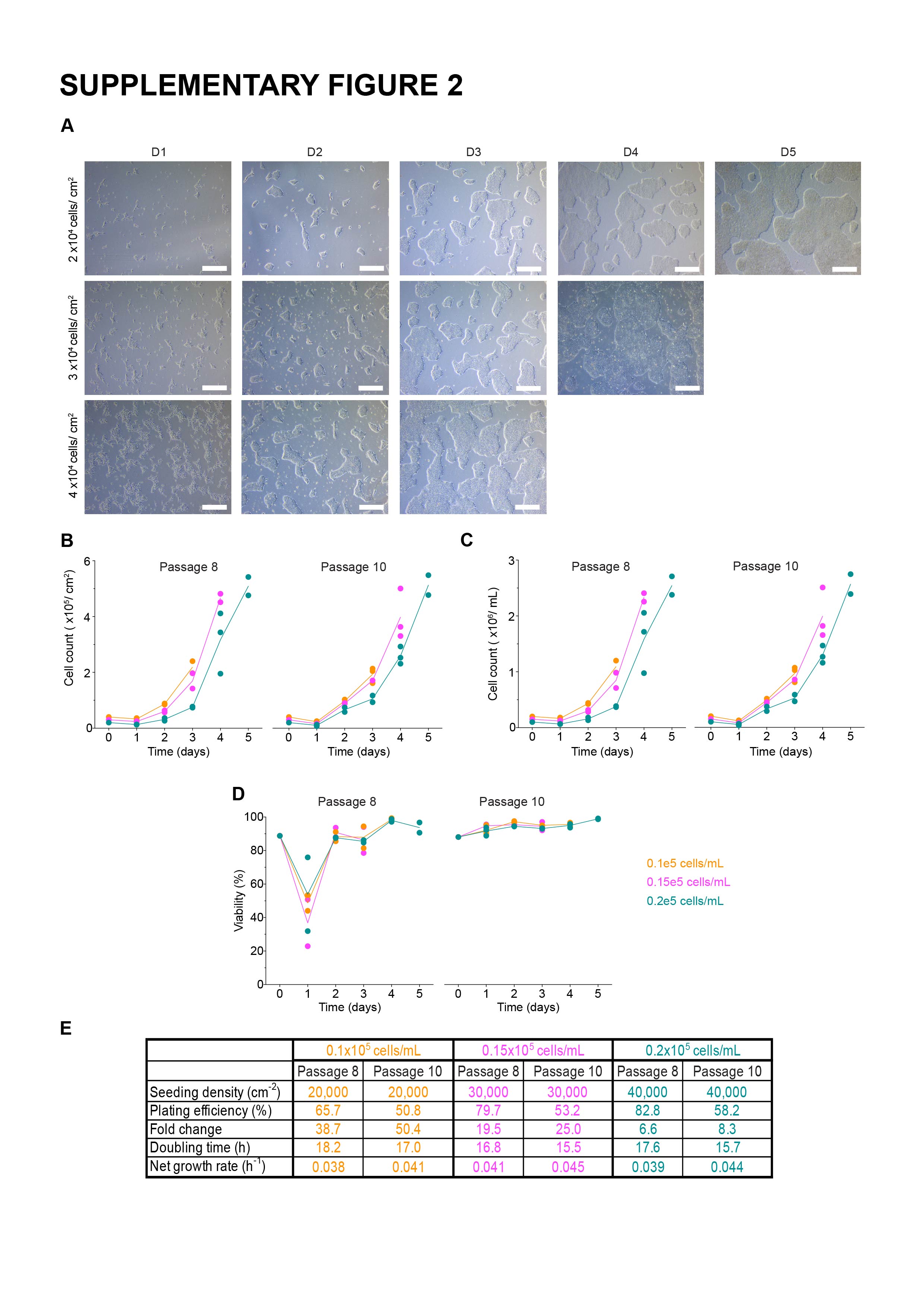

Supplement: Supplementary file 6 [file Image2.JPEG]

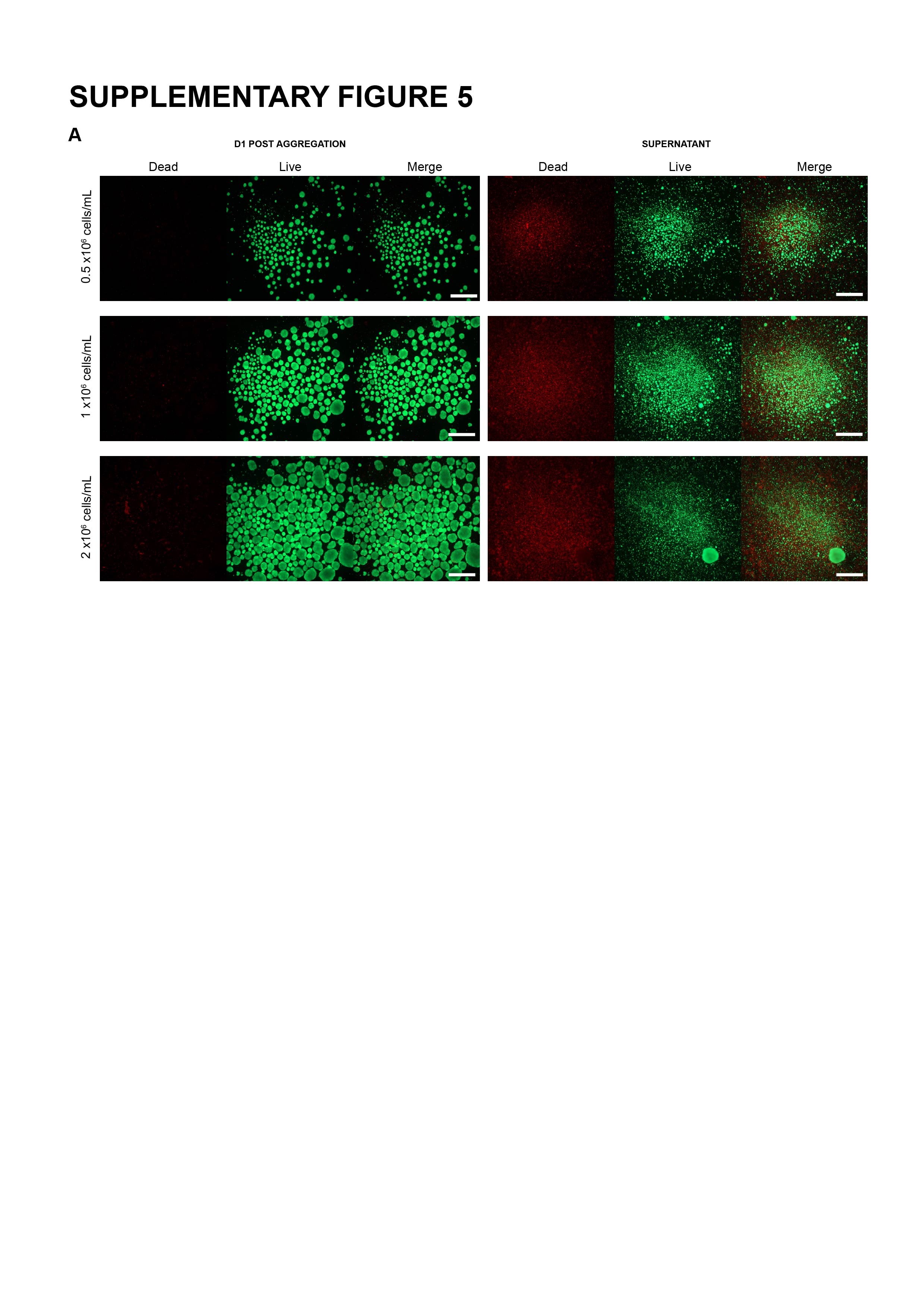

Supplement: Supplementary file 7 [file Image5.JPEG]

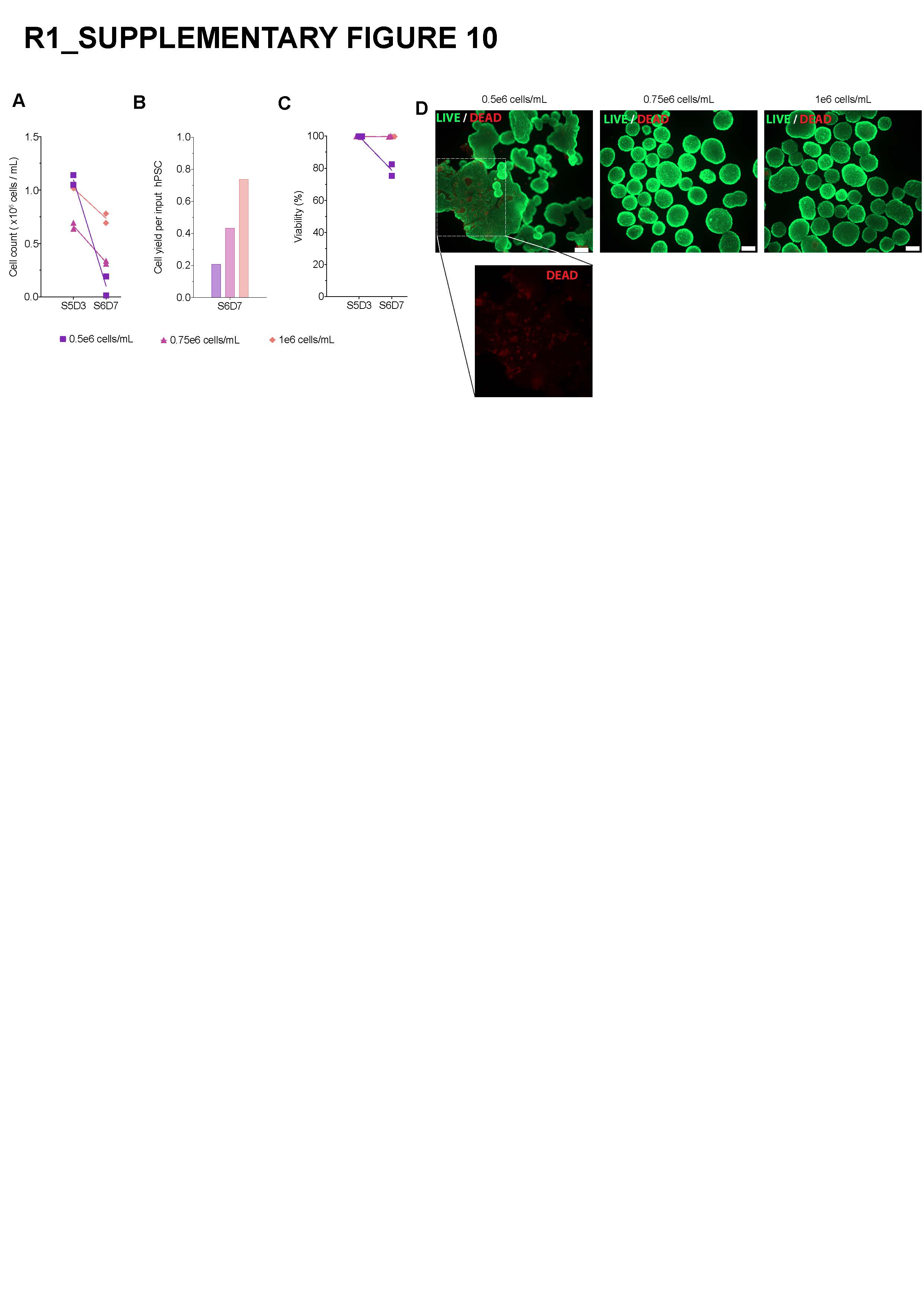

Supplement: Supplementary file 8 [file Image10.JPEG]

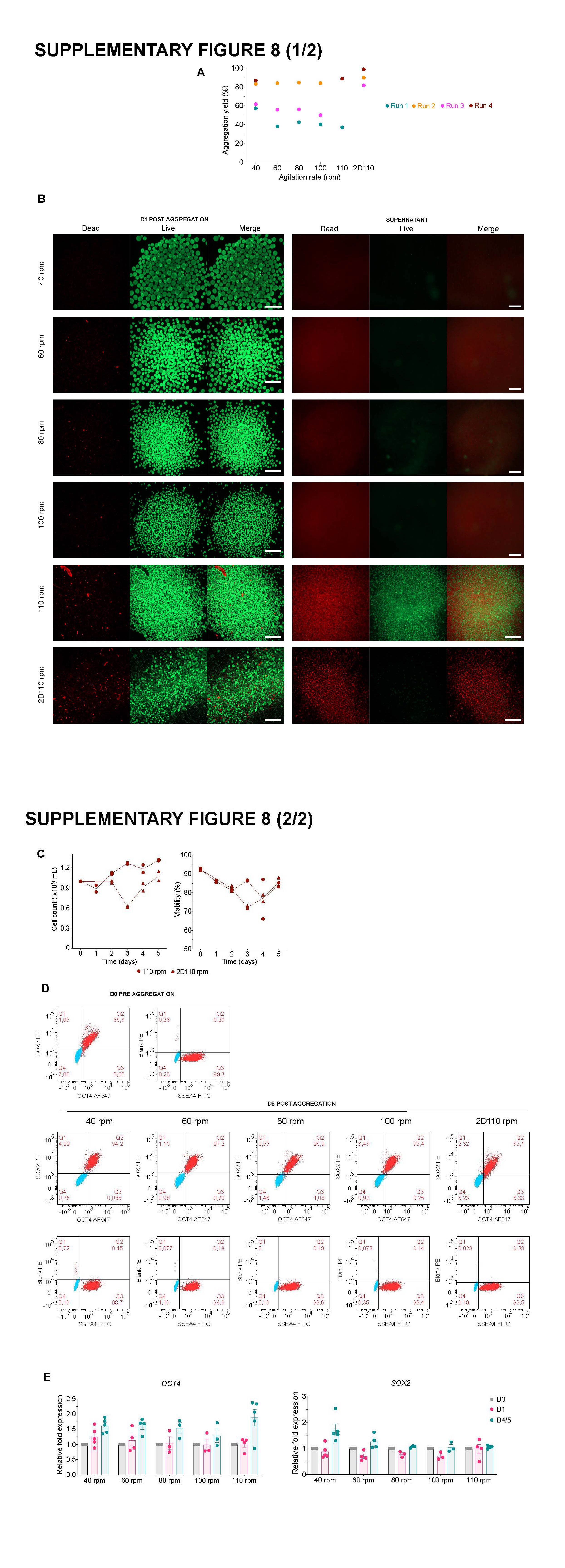

Supplement: Supplementary file 10 [file Image8.JPEG]

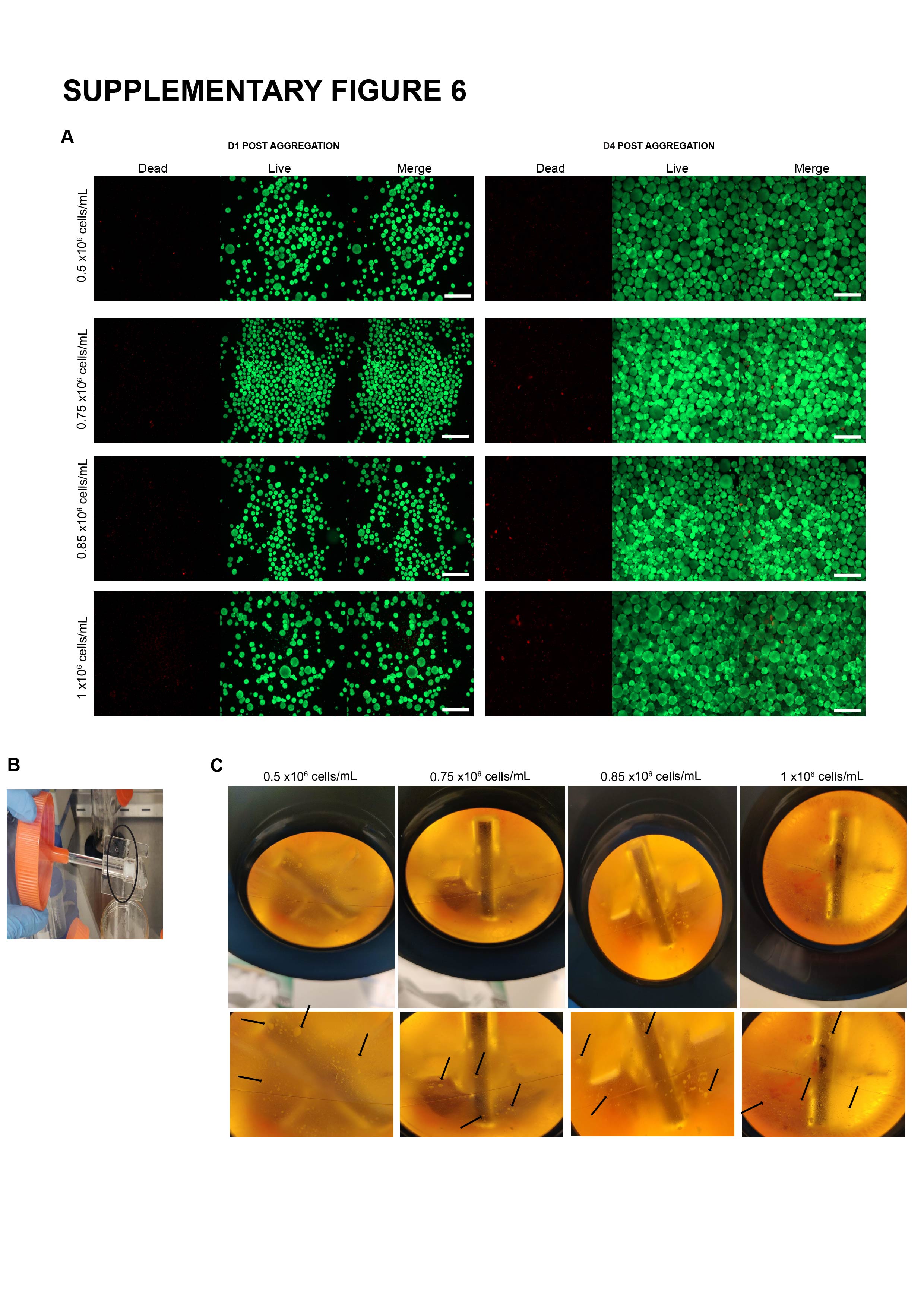

Supplement: Supplementary file 11 [file Image6.JPEG]
